# The impact of a maternal and offspring obesogenic diet on daughter’s oocyte mitochondrial ultrastructure and bioenergetic responses. Insights from an outbred mouse model

**DOI:** 10.3389/fphys.2023.1288472

**Published:** 2023-10-25

**Authors:** Inne Xhonneux, Waleed F. A. Marei, Ben Meulders, Silke Andries, Jo L. M. R. Leroy

**Affiliations:** ^1^ Department of Veterinary Sciences, Laboratory of Veterinary Physiology and Biochemistry, Gamete Research Centre, University of Antwerp, Wilrijk, Belgium; ^2^ Department of Theriogenology, Faculty of Veterinary Medicine, Cairo University, Giza, Egypt

**Keywords:** DOHaD, intergenerational diseases, oocyte quality, high-fat diet, maternal obesity, mitochondrial quality

## Abstract

Obesity affects oocyte mitochondrial functions and reduces oocyte quality and fertility. Obesity may also increase the risk of metabolic disorders in the offspring. Children are likely to follow their parents lifestyle and diet, which also contributes to the increased prevelance of obesity across generations. We hypothesise that the impact of obesogenic (OB) diet and obesity on oocyte mitochondrial functions is different in offspring born to obese mothers compared to those born to healthy mothers. To test this hypothesis, we fed a control (C, 10% fat, 7% sugar) or an OB diet (60% fat, 20% sugar) to female mice (for 7 weeks (w)) and then to their female offspring (for 7w after weaning) in a 2 × 2 factorial design (C » C, n = 35, C » OB, n = 35, OB » C n = 49 and OB » OB, n = 50). Unlike many other studies, we used an outbred Swiss mouse model to increase the human pathophysiological relevance. Offspring were sacrificed at 10w and their oocytes were collected. Offspring OB diet increased oocyte lipid droplet content, mitochondrial activity and reactive oxygen species (ROS) levels, altered mitochondrial ultrastructure and reduced oocyte pyruvate consumption. Mitochondrial DNA copy numbers and lactate production remained unaffected. Mitochondrial ultrastructure was the only factor where a significant interaction between maternal and offspring diet effect was detected. The maternal OB background resulted in a small but significant increase in offspring’s oocyte mitochondrial ultrastructural abnormalities without altering mitochondrial inner membrane potential, active mitochondrial distribution, mitochondrial DNA copy numbers, or ROS production. This was associated with reduced mitochondrial complex III and V expression and reduced pyruvate consumption which may be compensatory mechanisms to control mitochondrial inner membrane potential and ROS levels. Therefore, in this Swiss outbred model, while offspring OB diet had the largest functional impact on oocyte mitochondrial features, the mitochondrial changes due to the maternal background appear to be adaptive and compensatory rather than dysfunctional.

## Introduction

Obesity, linked to a sedentary lifestyle and an unbalanced diet, is a globally increasing problem leading to disappointing fertility. Obesity represents a major health issue associated with cardiovascular diseases, metabolic syndrome, insulin resistance and fatty liver disease among other metabolic disorders (Keller and Lemberg, 2003; Blondeau et al., 2011; Cardozo et al., 2011; Jialal et al., 2014; Johns et al., 2015; Broughton and Moley, 2017; Liu et al., 2021; Gonzalez et al., 2022). Almost 60% of women in Europe are overweight or obese. Furthermore, a steep increase is also seen in the proportion of children suffering from the same condition. About 30% of pregnant women suffer from obesity, which is known to increase the risk of offspring health issues (Armitage et al., 2008; Gahagan et al., 2012; Gaillard, 2015) such as metabolic syndrome, obesity and diabetes (Armitage et al., 2008; Bansal and Simmons, 2018). This could be replicated in mouse models fed an obesogenic (OB) diet during pregnancy while offspring were kept on a standard diet after weaning (Samuelsson et al., 2008; Igosheva et al., 2010; Jungheim et al., 2010; Keleher et al., 2018; Andreas et al., 2019; McMurray et al., 2019). While there is an increasing number of studies that describe an impact of maternal obesity on offspring health, the consequences for offspring fertility remain largely underexplored.

A key factor in the pathogenesis of subfertility linked to metabolic health disorders is a directly affected oocyte (Robker, 2008; Cardozo et al., 2011; Antunes et al., 2014; Leary et al., 2015). Obesity reduces oocyte quality mainly by inducing lipotoxicity. Lipotoxicity is defined as lipid accumulation in non-adipose tissue (Engin, 2017), causing inflammatory responses and oxidative stress, resulting in deleterious effects such as mitochondrial dysfunction (Wu et al., 2010; Van Hoeck et al., 2013; Leroy et al., 2022). Obesity alters the lipid composition of the ovarian follicular fluid, the microenvironment in which oocyte growth and maturation takes place. This is associated with intracellular accumulation of reactive oxygen species, altered mitochondrial membrane potential, lower mitochondrial mass and less available ATP, affecting not only cellular bioenergetics in the oocyte but also in the subsequent embryo, since mitochondria are exclusively maternally inherited. Mitophagy was shown not to be activated in oocytes in response to mitochondrial dysfunction, suggesting that dysfunctional mitochondria may be transmitted to the embryo (Boudoures et al., 2017c). This will not only lead to subfertility in the affected patient, but can also lead to mitochondrial aberrations transmitted to the offspring, as claimed by Saben et al. (2016) in inbred C57BL/6 mice. More mitochondrial ultrastructural abnormalities were detected in oocytes and muscles of adult offspring born to diet-induced obese C57BL/6 mothers, suggesting a transmission of aberrant mitochondria through the female germline (Saben et al., 2016). On the other hand, other studies show evidence of clearance of damaged mitochondria in the embryos during the peri-implantation period. At this stage, the so called bottleneck phenomenon only allows embryonic cells with healthy mitochondria to survive, which is suggested to minimize the transfer of defective mitochondria to the offspring (Lee et al., 2012; Cox et al., 2021). Of course, it can also be assumed that *de novo* mitochondrial dysfunction may take place in the offspring during prenatal and/or early postnatal development (during lactation) under obesogenic (OB) conditions.

In either cases, transmission or appearance of aberrant mitochondria in the early embryo and eventually the offspring, may directly influence metabolism and growth, and may render this next-generation more sensitive or vulnerable to lipotoxic conditions upon consuming an OB diet. However, it may also induce many indirect effects on the developing embryo or the offspring, through alterations in epigenetic programming (Desmet et al., 2016). Oocyte maturation under lipotoxic conditions changes mitochondrial functions and DNA methylation in the resulting preimplantation embryo (Meulders et al., 2022; Taschereau et al., 2023). This can result in epigenetic modifications in imprinted and developmentally important genes, affecting implantation, placentation, fetal development and compromising postnatal health (Dearden and Ozanne, 2015). On top, children born to obese mothers are more likely to develop the same dietary preference, which may lead to an increased preference to an OB diet with a higher risk of developing metabolic disorders and other complications such as infertility (Keleher et al., 2018; Pfledderer et al., 2021). However, the additive effect of the interaction between maternal and offspring OB conditions on the offspring’s oocyte quality, to the best of our knowledge, has not been studied yet.

Therefore we hypothesized that the impact of an OB diet on oocyte quality and mitochondrial function is influenced by the offspring’s maternal metabolic background. We aimed to study the influence of a maternal OB background on the effect of an offspring OB diet on oocyte quality and mitochondrial functions. For this an outbred mouse model was used in a two-by-two factorial design. We investigated the effect of 1) an offspring OB diet and/or 2) a maternal OB background and 3) the interaction between offspring and maternal diet, on offspring oocyte mitochondrial features. Our analyses were focused on oocyte lipid content and on oocyte mitochondrial qualitative and quantitative measures, including mitochondrial bioenergetic activity, morphology and cell metabolism.

## Materials and methods

### Animal model and experimental design

This study was approved by the Ethical Committee for Animal Testing (ECD 2018-05). All animal procedures were performed in accordance with the relevant guidelines and regulations.

We worked with outbred mice because inbred control fed C57BL/6 mice exhibit already a high rate of mitochondrial ultrastructural abnormalities in their oocytes (Marei et al., 2020) which may create a bias if used to answer the research questions listed above. A total of 26 female Swiss mice (F_0_) and a total of 169 of their female offspring were used in this study. The mothers were either fed a control diet (“C”, n = 11, Sniff diets D12450J, containing 10 kJ% fat and 7% sucrose (E157453-04)) or an OB diet (n = 15 (E15741-34), 60 kJ% fat (beef Tallow), 20% fructose adjusted in the drinking water) for 7 weeks starting at 3 weeks of age. Mothers were weekly weighed for 7 weeks of being fed the corresponding diet. Detailed information on maternal weight is included in Supplementary File S1. All females were mated with Swiss males (n = 4) fed a standard chow diet, in a cross over design (i.e., each male is used in both groups). Litter sizes were equalised at 10 pups, to correct for differences in nutrient availability from postnatal day 5–22 to equalize establishment of maternal imprints. Detailed information on litter size is included in Supplementary File S1. Female F_1_ offspring from each litter were equally divided at weaning on a C or OB diet creating a 2 × 2 factorial study design, ultimately resulting in 4 treatment groups: 1) C » C, n = 35 offspring born to control mothers and fed a control diet, 2) C » OB, n = 35 offspring born to control mothers, and fed an OB diet 3) OB » C, n = 49 offspring born to obese mothers and fed a control diet 4) OB » OB, n = 50 offspring born to obese mothers and fed an OB diet. Offspring were weaned at 3 weeks and were fed their corresponding diet for 7 weeks afterwards. Adult offspring were weighed weekly and sacrificed by decapitation at 10 weeks of age after intraperitoneal injection of 10IU PMSG (Pregnant Mare Serum Gonadotropin, Folligon 1000UI, MSD Intervet, Boxmeer, Netherlands) and 10IU hCG (human choriogonadotropin, Pregnyl 5000I.E., MSD Intervet), respectively. Decapitation was quickly performed by trained personnel (FELASA C) using a sharp blade. Blood was collected for total serum cholesterol, enzymatically analyzed (A.M.L., Antwerp, Belgium) on an Abbott Architect c16000 (Abbott, Illinois, U.S.A) as described by (Allain et al., 1974; Roeschla et al., 1974). Cumulus-oocyte complexes (COCs) were collected directly from the oviduct, i.e., after *in vivo* maturation and ovulation, immediately after euthanasia as described by Marei et al. (2020). One COC from each offspring was fixed in 2.5% glutaraldehyde solution for transmission electron microscopy (TEM). The remaining COCs were denuded of cumulus cells using stripper tips fitted on EZ-grip (Origio) in droplets of L15 medium (Thermofisher Scientific), supplemented with 50IU/mL Penicillin G Sodium salt, 0.3 mg/mL hyaluronidase and 10% Fetal Bovine Serum. Oocytes were denuded and used directly or after fixation (as described in the methods sections) to determine the lipid droplet content (LDC), the mitochondrial inner membrane potential (MMP), the distribution of the active mitochondria and the reactive oxygen species (ROS) accumulation. The mitochondrial DNA copy number was assessed using qPCR and the oxidative phosphorylation (OXPHOS) was estimated by assessing electron transport chain (ETC) complex markers. The lactate production and pyruvate consumption of the oocytes was analysed to estimate the cellular metabolic activity.

### Assessment of oocyte lipid droplet content

Lipid droplet content (LDC) was assessed using BODIPY^®^ staining and confocal microscopy. Offspring from 7 to 8 C mothers and 6-8 OB mothers were used for the analysis of the oocyte LDC. At least 5 oocytes per offspring (n = seven to eight offspring per group) were fixed in paraformaldehyde 4% and stored in PBP containing 1 mg/mL PVP (PBS-PVP) at 4°C for a maximum of 1 month. Oocytes were permeabilized in PBS containing saponin (0.1%w/v) and glycine 0.1 M solution, then washed and incubated in 20 μg/mL BODIPY (BODIPY^®^ 496/503, Thermo Fisher Scientific, Belgium) in PBS for 1 h. The presence of a polar body was included in the analysis. After each step, oocytes were washed twice in PBS-PVP. Oocytes were mounted in glass-bottom dishes in droplets of PBS-PVP and immediately examined using confocal microscope (Nikon Eclipse Ti-E inverted micro-cope attached to a microlens-enhanced dual spinning disk confocal system (UltraVIEW VoX; PerkinElmer, Zaventem, Belgium)). High resolution images were acquired with 488 nm diode lasers, excitation 493 nm and 503 nm emission. Z-stack projections of 40 µm with 1 µm steps were taken for each oocytes. The volume of lipid droplets was measured using Volocity^®^ software (Quorum Technologies Inc., Puslinch, Canada). To exclude background noise, objects smaller than 0.5 μm^3^ were excluded from the analysis. Finally, lipid volume per oocyte was calculated based on the oocyte diameter.

### Assessment of oocyte mitochondrial DNA copy number

DNA extracts from pools of at least 15 oocytes per litter from 11 C to 13 OB mothers, were used to determine the absolute amount of mitochondrial DNA qPCR of the mitochondrial gene ND4. After simultaneous purification of genomic and total RNA using AllPrep^®^ DNA/RNA MicroKit (#80284, Qiagen) and CYBR Green (SYBR Green supermix #172-5270, Bio-Rad) was mixed with 20 pmol/μL forward and reverse primers in nuclease-free water and sample DNA. The absolute amount of mitochondrial DNA was measured using Avogadro’s constant as “Number of copies=(ng * [6.022*10^23^])/(length*[1*10^9^]*650)” in a standard curve with known concentration and copies of ND4 in Bio-Rad CFX Manager 3.1.

### Assessment of mitochondrial inner membrane potential, reactive oxygen species accumulation and distribution of active mitochondria

One offspring of each of the 6–8 C and 6-8 OB mothers was used to analyse the MMP, the active mitochondrial distribution and accumulation of reactive oxygen species (ROS), using specific staining and confocal microscopy. Only mature oocytes, with a polar body, were used for this assessment. Six to eight oocytes per offspring were incubated for 30 min at 6% CO_2_ 37°C in L15 medium containing 5 μg/mL 5,5′,6,6′-tetrachloro-1,1′,3,3′-tetraethylbenzimidazolyl-carbocyanine iodide (JC-1, Invitrogen) and 2.5 mM CellROX™ Deep Red Reagent (Thermo Fisher Scientific, Belgium) as described by Komatsu et al. (2014) and De Biasi et al. (2015) directly after collection of the oocytes. i.e., without a fixation step. Afterwards, oocytes were washed and transferred to equilibrated L15 medium droplets under mineral oil on 35 mm glass-bottom dishes. Images were taken using LeicaSP8 laser scanning confocal microscope (Leica Microsystems, Machelen, Belgium) in a humid chamber at 37°C. The uptake of JC-1 and formation of J-aggregates in the mitochondria is MMP dependent and hence used to estimate mitochondrial bioenergetic activity. Images from three confocal planes from each oocyte: the equatorial plane (at largest diameter), the peri-cortical plane (closest to the objective lens), and plane in-between) were acquired at excitation/emission 488/525 nm (monomers, mitochondria with low MMP or a reduced activity; green), 561/590 nm (formation of J-aggregates with high MMP, active mitochondria; yellow) and 644/665 nm (to detect CellROX or accumulation of ROS; red). The mean grey scale intensity of the MMP and ROS was afterwards quantified using ImageJ software. Active mitochondria are reported as mean grey scale intensity of the J-aggregates. Inactive mitochondria are reported as the mean grey scale intensity of the JC-1 monomers. The presence and thickness of the active mitochondrial ring in the peri-cortical region was measured using ImageJ and used to categorize the regional distribution of active mitochondria as peri-cortical or diffuse. Mitochondria showing no activity were categorized as uncoupled and mitochondrial aggregates were classified as aberrant clustering.

### Assessment of mitochondrial ultrastructure in oocytes

One COC per offspring from 8 C to 8 OB mothers was fixed upon collection in 0.1 M sodium cacodylate-buffered (pH 7.4) 2.5% glutaraldehyde solution, embedded in 2% agarose blocks and washed in 0.1 M sodium cacodylate-7.5% saccharose solution (pH 7.4). Then the blocks were incubated for 2 h in 1% OsO_4_ solution and dehydrated in an ethanol gradient. Sections were cut ultrathin using EM-bed812, stained with lead citrate and examined with transmission electron microscope Tecnai G2 Spirit Bio TWIN microscope (Fei, Europe BV, Zaventem, Belgium) at 120 kV. For each oocyte, 10-20 random images were acquired at ×16500 magnification. Mitochondria were morphologically evaluated and classified blindly according to (Marei et al., 2020).

### Assessment of mitochondrial electron transport chain complexes

Pools of 45–80 oocytes of 2-5 offspring from 3 to 4 C and OB mothers were used to analyse the expression of the ETC, complex markers for Western blotting (WB) after collection. Different complex markers of the ETC, were analysed (from 2-5 offspring per mother), using total OXPHOS Rodent WB Antibody Cocktail (Abcam, ab110413) and GAPDH Antibody (Thermo Fisher Scientific™, US, PA1-16777) as a housekeeping protein for relative quantification. After lysis of denuded oocytes in lysis buffer containing Trizma^®^ hydrochloride solution (Sigma-Aldrich, US, T3038, 63.5 mM), Glycerol (Sigma-Aldrich, G7893, 10% v/v), sodium dodecyl sulphate (Sigma-Aldrich, L5750, 4% w/v) and protease (Thermo Fisher Scientific™, 78,425)-phosphatase (Thermo Fisher Scientific™,78,420) inhibitor at pH 6.8, followed by freeze-thaw cycles, SDS-page electrophoresis was performed on mini PROTEAN pre-cast gels (Bio-rad, TGX Precast Protein Gels, 4561021) at 170 V and 0.5 A for 70 min. Proteins were transferred on PVDF-membranes at 50 V and 0.1 A for 1 h 35 min and blocked for 2 h in 0.1% Skimmed milk (Applichem, A0830). After overnight blotting with OXPHOS Rodent kit (dilution 1:500), Goat-anti-Mouse biotinylated antibody (dilution 1:2000, Dako E0433) was used as secondary antibody, followed by Streptavidine-HRP (dilution 1:2000, Dako P0397) blotting. As a last step, LumiGLO™ (Cell Signalling, 7003S) was utilized and images were acquired and analysed with Chemidoc XRS + Gel Documentation System (BioRad, Belgium). Antibodies were dissolved in 10% bovine serum albumin (Sigma, S2002) solution containing 0.1% of NaN_3_. In between different blotting steps, the membrane was washed with TBST-Tween solution for 2 × 5 minutes, followed by 20 min of washing. Negative controls, without the primary antibody, were included for validation.

### Assessment of oocyte lactate production and pyruvate consumption

Six to 8 freshly collected mature oocytes of 1 offspring coming from 8 C to 8 OB mothers were immediately incubated for 4 h in 7 µL droplets of L15 lactate-free 5% CO_2_ and 5% O_2_ equilibrated medium containing 0.5 mM pyruvate under mineral oil. The fluorometric assay is based on the generation or consumption of the reduced pyridine nucleotides, NADH and NADPH, in coupled enzymatic reactions. The nucleotides fluoresce when excited at 340 nm, whereas the oxidized forms, NAD^+^ and NADP^+^, do not. The fluorometric conversion of NADH to NAD^+^ (or *vice versa*) was measured with TECAN Infinite 200Pro (TECAN Group Ltd., Männedorf, Switzerland) after adding lactate dehydrogenase (Roche, 10127876001 5 mg/mL, 1,100 U/mg), with or without adding hydrazine (Sigma, H3376) (Gardner D.K. and Lane M., 2013).

### Statistical analysis

Statistical analysis was done using IBM SPSS Statistics 28 (for Windows, Chicago, IL, United States). Numerical data (e.g., LDC, MMP, ROS, OXPHOS, lactate production and pyruvate consumption) were checked for equality of variance (Levene’s Test) and normality of distribution (residual QQ-plots). The effect of the maternal diet, the offspring diet and their interactions were investigated using Two-way ANOVA. If the interaction was not significant, the interaction was omitted from the model. Comparisons of the two offspring dietary groups within each maternal group, and *vice versa*, were done using Independent Samples *T-tests* (summarised in Supplementary File S2). In all models, the maternal dietary groups and the offspring dietary groups were used as fixed factors, while the mother ID was used as a random factor if two or more sisters were present in the same offspring treatment group (e.g., offspring weight). Categorical data (e.g., proportions of different distribution patterns of the active mitochondria and ultrastructural classifications) were analysed using general linear mixed models (binary logistic regression). Pairwise comparisons within each offspring or maternal group were done using Chi-square analysis or Fisher’s Exact analysis when the expected frequency was below five (e.g., mitochondrial clustering and uncoupling (Supplementary File S2)). The differences in mean weight data recorded weekly throughout the experiment were analysed using repeated measures ANOVA, and Two-way ANOVA measurements were performed to study the effect of maternal diet and offspring diet on offspring mean weight at each timepoint. Correlations between offspring body weight and oocyte outcome parameters were done when no oocyte pooling between offspring and litters was needed to acquire sufficient sample size (i.e., this was done for JC-1 and CellROX™ data, metabolic assay measurements and ultrastructural abnormalities only).

## Results

In all results sections below, the results of main effects using Two-way ANOVA or binary logistic regression are reported first to show the effects of maternal diet, offspring diet and their interaction. This is followed by analysing maternal effects in pairwise comparison within each offspring diet group, or *vice versa*. A summary of the main effects and pairwise comparisons of all outcome parameters are shown in Supplementary Table S2 of the Supplementary File S2. Results of the maternal diet effects on mother weight and litter size are implemented in Supplementary File S1.

### Offspring live body weight and total cholesterol

**FIGURE 1:**
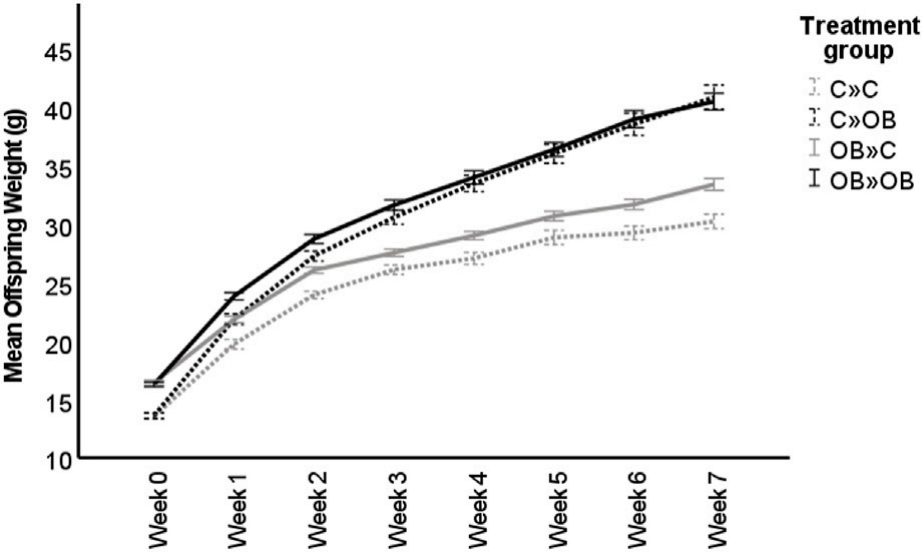
Maternal and offspring diet effects on offspring weight trajectory. The graph shows the live body weight trajectory (g) in offspring fed a control (C) or an obesogenic diet (OB) and born to mothers that were either fed a C or OB diet, in a 2 × 2 factorial design. The groups are named as MaternalDiet » OffspringDiet. Data are presented as mean ± S.E.M. and are derived from 11 litters of C mothers and 15 litters of OB mothers.

The body weight trajectory was significantly affected by diet, time, and the interaction between both factors (*p* < 0.05, Figure 1).

The effect of the offspring obesogenic diet on offspring body weight was significant already after 1 week of dietary exposure and was further significant until sample collection at 10 weeks of age (*p* < 0.05, Table 1). Maternal obesogenic diet significantly increased offspring body weight at week 1 and week 2 post-weaning, but disappeared from week 3 onwards (*p* > 0.05, Table 1). An interaction between maternal and offspring diet effects was only present after being fed the corresponding diet for 7 weeks (*p* < 0.05, Table 1). Offspring body weight was not correlated with litter size (r = 0.015, *p* < 0.05).

**TABLE 1:** Offspring weekly weights of C- or OB-fed offspring and born to mothers that were either fed a C or OB diet, in a 2 × 2 factorial design. Data are presented as mean ± S.E.M. and are derived from 40 to 41 pups of 11 C mothers and 56-57 pups from 15 OB mothers. *p-*values of the main effects are stated (F_0_ = maternal diet effect, F_1_ = offspring diet effect, I = interaction).

	Week 0 (weaning, g)	Week 1 (g)	Week 2 (g)	Week 3 (g)	Week 4 (g)	Week 5 (g)	Week 6 (g)	Week 7 (g)
C » C	13.55 ± 0.24	19.74 ± 0.41	23.96 ± 0.32	26.12 ± 0.40	27.09 ± 0.51	28.87 ± 0.61	29.27 ± 0.59	30.25 ± 0.61
C » OB	13.62 ± 0.24	21.88 ± 0.46	27.28 ± 0.43	30.60 ± 0.60	33.50 ± 0.70	36.04 ± 0.81	38.57 ± 0.95	40.90 ± 1.04
OB » C	16.43 ± 0.20	21.85 ± 0.28	26.08 ± 0.27	27.57 ± 0.32	29.04 ± 0.36	30.71 ± 0.41	31.70 ± 0.44	32.92 ± 0.50
OB » OB	16.28 ± 0.21	23.84 ± 0.31	28.75 ± 0.39	31.65 ± 0.44	34.02 ± 0.57	36.41 ± 0.60	39.01 ± 0.73	40.52 ± 0.73
F_0_ *P*	0.000	0.034	0.006	0.110	0.174	0.286	0.198	0.332
F_1_ *P*	0.518	0.000	0.000	0.000	0.000	0.000	0.000	0.000
I *P*	0.691	0.744	0.328	0.727	0.163	0.194	0.135	0.025

When splitting the offspring based on their maternal metabolic background, body weights of both C-born and OB-born offspring were increased when fed an OB diet compared to the C-fed mice in the same subgroup. Interestingly, although the two-way ANOVA showed no maternal diet effect, only control offspring from OB mothers were significantly heavier compared to control offspring born to control mothers. A significantly higher weight could be detected in offspring born to OB mothers only when the offspring were fed a control diet (32.92 ± 0.5 g in OB » C vs. 30.25 ± 0.61 g in C » C, *p* < 0.05), but not in offspring fed an OB diet (40.90 ± 1.04 g in C » OB vs. 40.52 ± 0.73 g in OB»0B, *p* > 0.05).

Offspring diet caused a significant increase in offspring total serum cholesterol (C » C 112.9 ± 8.5 vs. C » OB 180.0 ± 5.4 and OB » C 126.9 ± 68.2 vs. OB » OB 193.0 ± 10.0, *p* < 0.05). Maternal diet and the interaction maternal x offspring diet showed no significant differences (*p* > 0.05). Pairwise comparison between offspring or maternal diet groups showed no significant differences (*p* > 0.05). Offspring total serum cholesterol was positively correlated with offspring body weight (r = 0.786, *p* < 0.05).

### Offspring oocyte lipid droplet content

**FIGURE 2:**
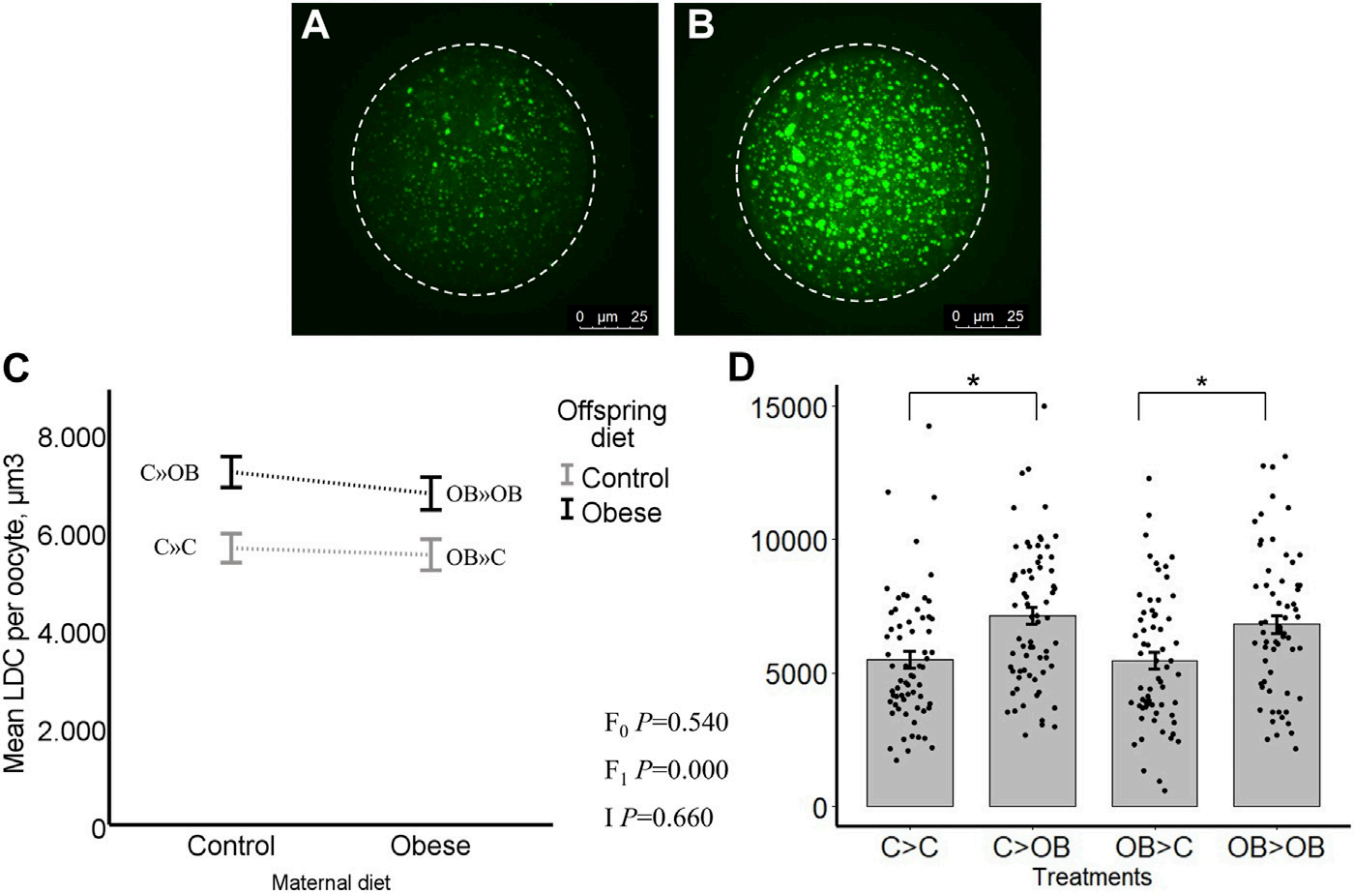
Maternal and offspring diet effects on offspring oocyte lipid droplet content (LDC). Representative confocal projections of oocytes stained with BODIPY^®^ showing lipid droplets (green) from the C » C **(A)** and the C » OB **(B)** groups. Graphs showing LDC (µm3/oocyte) in oocytes collected from offspring fed a C or an OB diet and born to mothers that were either fed a C or OB diet, in a 2 × 2 factorial design using an interaction plot **(C)** and a dot plot **(D)**. Data are presented as mean ± S.E.M. and are derived from 5 to 10 oocytes per litter per group, from 6-7 litters or mothers. *p*-values of the main effects are stated (F_0_ = maternal diet effect, F_1_ = offspring diet effect, I = interaction). Significant differences are indicated with an asterisk (*), tendencies with a dollar sign ($). Pairwise comparisons within treatment groups are presented in Supplementary File S2.

The LDC was significantly higher in oocytes collected from offspring fed an OB diet (*p* < 0.05) regardless of their maternal background. The LDC was significantly increased in C » OB compared to C » C (7.2 ± 0.3 vs. 5.7 ± 0.3, x10^2^ μm^3^) and in OB » OB vs. OB » C (6.8 ± 0.3 vs. 5.5 ± 0.3, x10^2^ µm^3^, Figure 2). Maternal diet had no effect, and there was no interaction between the maternal and offspring diet effects (*p* > 0.05). The presence of a polar body did not influence the LDC of the oocytes and did not influence the effect of diet on LDC (separate data not shown).

### Offspring oocyte mitochondrial DNA copy number

**FIGURE 3:**
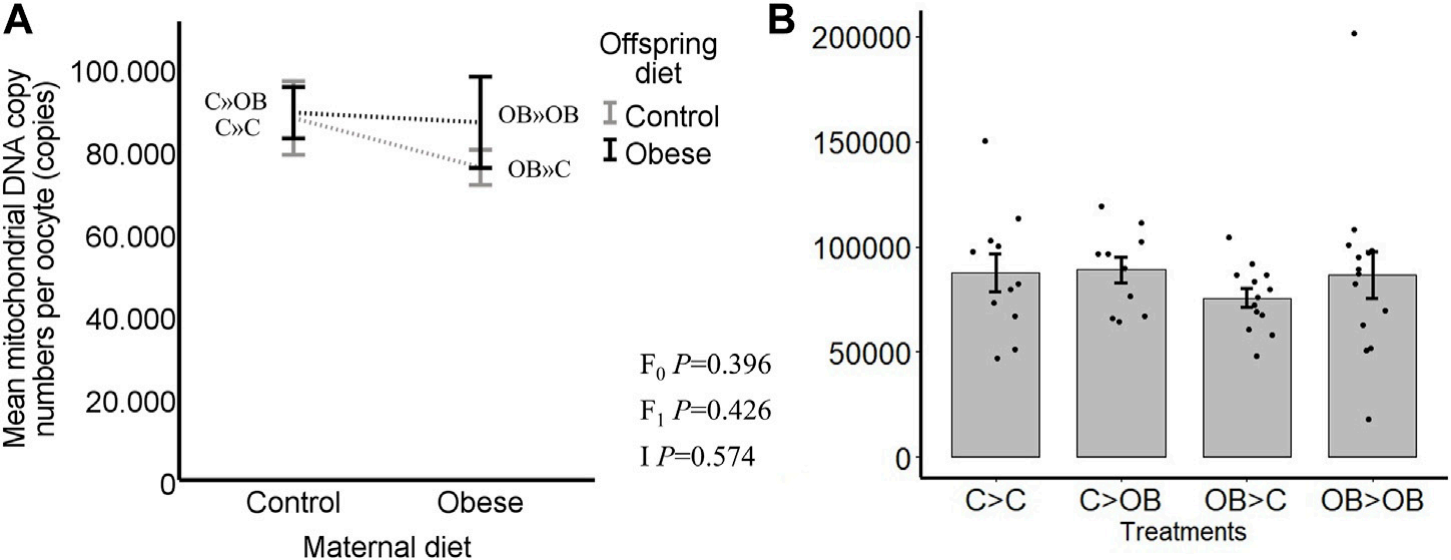
Maternal and offspring diet effects on offspring oocyte mitochondrial DNA copy number. Graphs representing mean mitochondrial DNA copy number (n copies/oocyte) in oocytes collected from C- or OB-fed offspring and born to mothers that were either fed a C or OB diet, in a 2 × 2 factorial design, using an interaction plot **(A)** and a dot plot **(B)**. Data are presented as mean ± S.E.M. and are derived from pools of at least 15 oocytes per group per litter, from 11 to 13 litters each. *p-*values of the main effects are stated (F_0_ = maternal diet effect, F_1_ = offspring diet effect, I = interaction). Significant differences are indicated with an asterisk (*), tendencies with a dollar sign ($). Pairwise comparisons within treatment groups are presented in Supplementary File S2.

Offspring oocyte mitochondrial DNA copy number was not affected by offspring diet, nor by maternal diet (C » C, 87.7 ± 8.9; C » OB, 89.0 ± 6.2; OB » C, 75.7 ± 4.3; OB » OB, 86.7 ± 11.1, x10^3^ copies/oocyte, *p* > 0.05, Figure 3). Pairwise comparisons within maternal diet groups or offspring diet groups showed no differences (*p* > 0.05).

### Offspring oocyte MMP, distribution of active mitochondria and ROS

**FIGURE 4:**
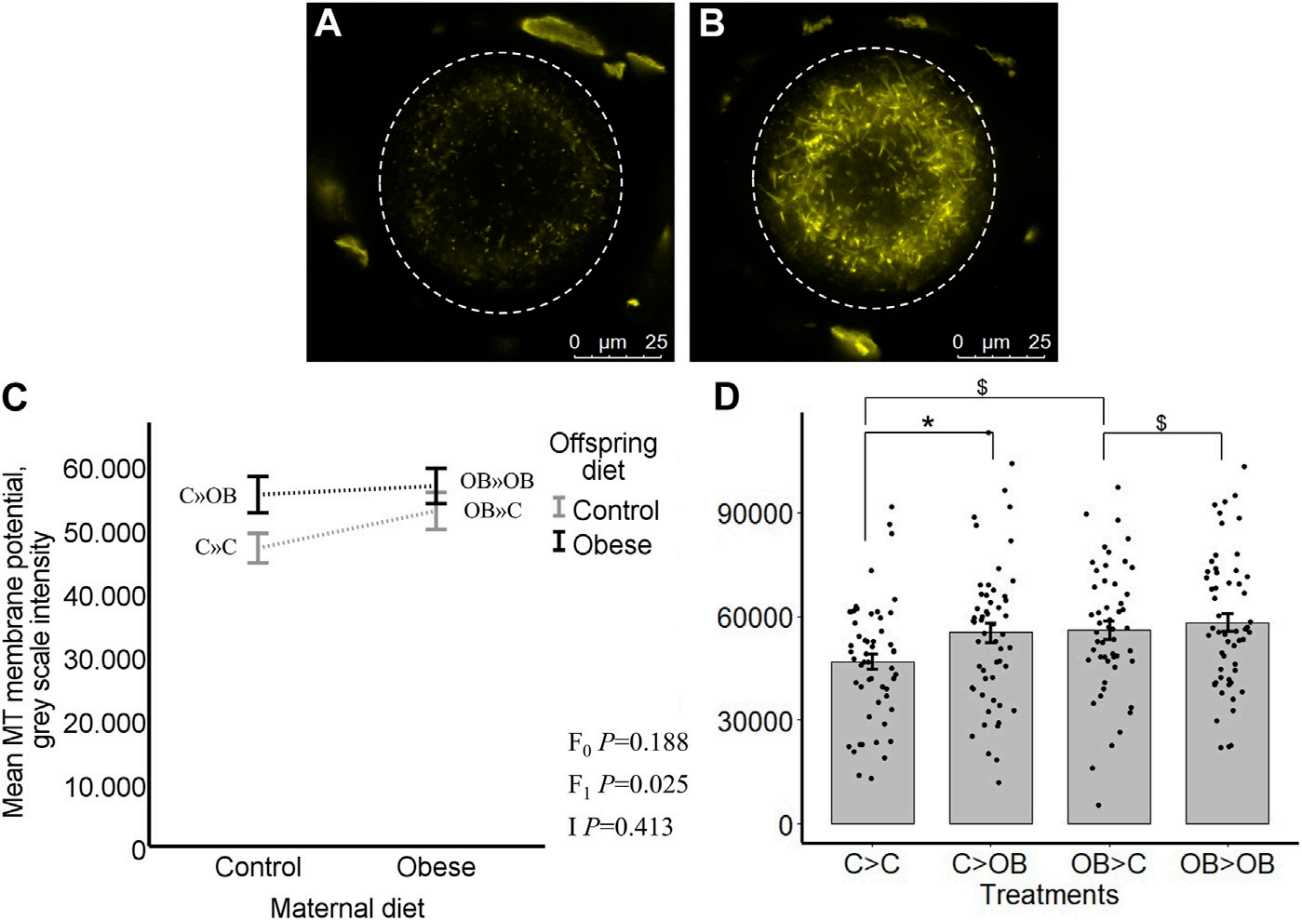
Maternal and offspring diet effects on offspring oocyte mitochondrial membrane potential (MMP). Representative confocal projections of oocytes stained with JC-1 (J-aggregates, yellow) from the C » C **(A)** and the C » OB **(B)** groups. Graphs representing mean MMP ((grey scale intensity)/oocyte) in oocytes collected from C- or OB-fed offspring and born to mothers that were either fed a C or OB diet, in a 2 × 2 factorial design, using an interaction plot **(C)** and a dot plot **(D)**. Data are presented as mean ± S.E.M. and are derived from 8 oocytes per offspring per group per litter, from 8 litters each. *p-*values of the main effects are stated (F_0_ = maternal diet effect, F_1_ = offspring diet effect, I = interaction). Significant differences are indicated with an asterisk (*), tendencies with a dollar sign ($). Pairwise comparisons within treatment groups are presented in Supplementary File S2.

Offspring oocyte MMP was only altered by offspring diet (*p* < 0.05). Maternal diet effect and maternal x offspring diet interaction were not significant (*p* > 0.05, Figure 4). In the pairwise comparisons within C mothers, C » OB mice had a significantly higher oocyte MMP compared to C » C (55.7 ± 2.9 vs. 46.9 ± 2.3 x 10^3^ grey scale intensity of J-aggregates, *p* < 0.05), while this increase was only a tendency in OB-born offspring (OB » OB, 56.3 ± 2.7 vs. OB » C, 52.9 ± 3.0,x10^3^ *p* < 0.1). In comparisons within each offspring diet group, maternal diet tended to affect MMP in oocytes from offspring fed a control diet (comparing OB » C *versus* C » C (*p* < 0.1)), whereas no differences occurred between OB » OB and C » OB (*p* > 0.05). Oocyte MMP was positively correlated with offspring body weight (r = 0.402, *p* < 0.05).

**FIGURE 5:**
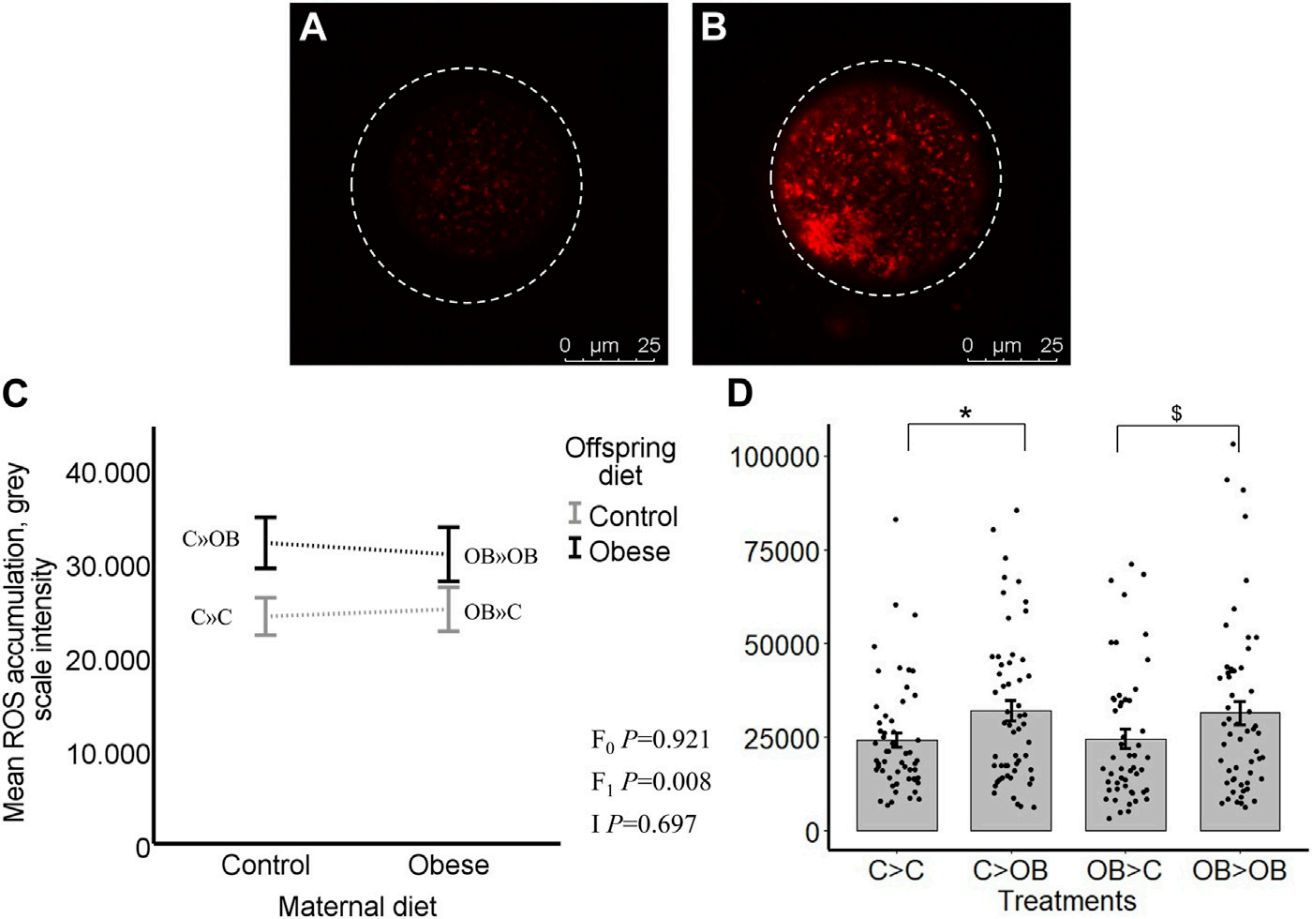
Maternal and offspring diet effects on offspring intraoocyte reactive oxygen species (ROS) accumulation. Representative confocal projections of oocytes stained with CellROX™ Deep Red from the C » C **(A)** and the C » OB **(B)** groups. Graphs showing intraoocyte ROS accumulation ((grey scale intensity)/oocyte) in oocytes collected from C- or OB-fed offspring and born to mothers that were either fed a C or OB diet, in a 2 × 2 factorial design, using an interaction plot **(C)** and a dot plot **(D)**. Data are presented as mean ± S.E.M. and are derived from 8 oocytes per offspring per group, from 8 litters or mothers. *p*-values of the main effects are stated (F_0_ = maternal diet effect, F_1_ = offspring diet effect, I = interaction). Significant differences are indicated with an asterisk (*), tendencies with a dollar sign ($). Pairwise comparisons within treatment groups are presented in Supplementary File S2.

Oocyte ROS levels (grey scale intensity) were only affected by offspring diet (*p* < 0.05). Maternal diet effect was not significant (*p* > 0.05, Figure 5). Despite no significant interaction, the increase in oocyte ROS levels due to offspring diet was only significant in C-born offspring (C » OB, 31.9 ± 2.6 vs. C » C 24.2 ± 2.0 × 10^3^, *p* < 0.05) and this was only a tendency (*p* < 0.1) in OB-born offspring (OB » OB, 30.6 ± 3 vs. OB » C, 25.3 ± 2.4, x10^3^). The ratios of ROS concentrations to active mitochondria (ROS: J-aggregates) were not affected by offspring nor maternal diet (separate data are not shown). Oocyte ROS accumulation was not correlated with offspring body weight (r = 0.061, *p* > 0.05).

**FIGURE 6:**
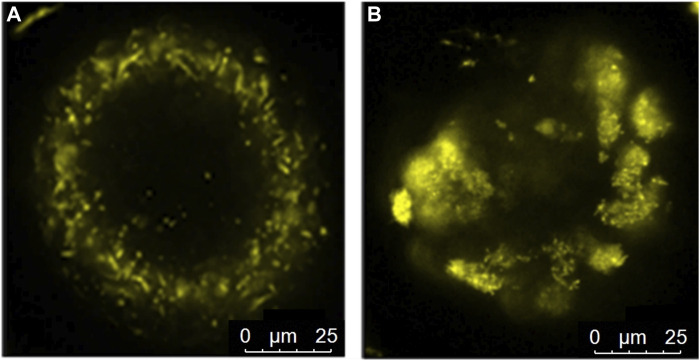
Peri-cortical distribution **(A)** of active mitochondria in an oocyte from the C » C group and mitochondrial clustering **(B)** in an oocyte from the OB » OB group.

The distribution of active mitochondria was neither affected by the offspring diet nor the maternal diet (*p* > 0.05). Nonetheless, a small fraction (6%–8%) of the oocytes of OB-born offspring showed either clustering of highly active mitochondria (Figure 6) or, on the other extreme, very low mitochondrial activity (uncoupling). This was associated with high intra-oocyte ROS accumulation. The proportions of oocytes exhibiting these defects were 6.25% ± 4.72% in OB » C and 8.71% ± 4.60% in OB » OB compared to 0% ± 0.0% in both C » C and C » OB (*p* < 0.05). The distribution of active mitochondria was not correlated with offspring body weight (r = −0.047, *p* > 0.05).

### Offspring oocyte mitochondrial ultrastructure

Oocyte mitochondrial ultrastructure was classified as normal when they were spherical and homogenous, with or without regular vacuoles. Mitochondria were classified as abnormal if they showed irregular vacuolated with or without loose membranous structures, dumbbell, elongated, or rose petal shape, electron dense foci or signs of degeneration (as described in the Supplementary Material in Marei et al., 2020).

**FIGURE 7:**
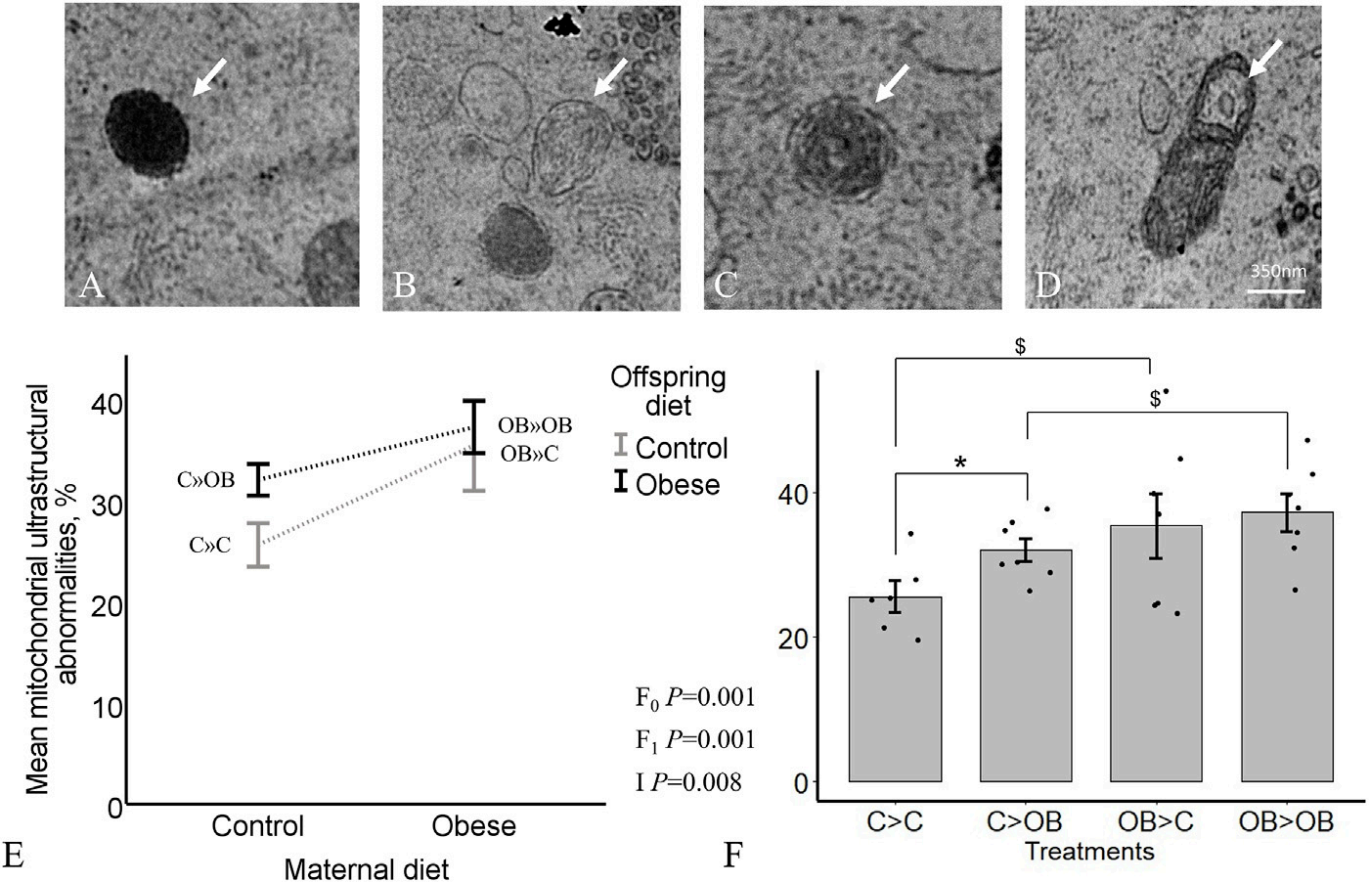
Maternal and offspring diet effects on offspring oocyte mitochondrial ultrastructure. Representative TEM images of oocytes showing electron dense foci **(A)**, degenerated **(B)**, rose-petal **(C)** and elongated mitochondria with loose inner membrane **(D)** are shown. The graphs represent mitochondrial ultrastructural abnormalities in oocytes collected from C- or OB-fed offspring and born to mothers that were either fed a C or OB diet, in a 2 × 2 factorial design, using an interaction plot **(E)** and a dot plot **(F)**. Data are presented as mean proportions ±S.E.M. and are derived from 1 oocyte per offspring per group, from 8 litters or mothers. *p-*values of the main effects are stated (F_0_ = maternal diet effect, F_1_ = offspring diet effect, I = interaction). Significant differences are indicated with an asterisk (*), tendencies with a dollar sign ($). Pairwise comparisons within treatment groups are presented in Supplementary File S2.

The proportions of oocyte abnormal mitochondria in total were significantly increased both by offspring and maternal OB diet (*p* < 0.05). In addition, maternal x offspring diet interaction was also significant (*p* < 0.05, Figure 7). This is because the increase in response to offspring OB diet was only significant in C-born offspring (32.11 ± 1.6 in C » OB vs. 25.7 ± 2.2 in C » C, *p* < 0.05), but was not significant in OB-born mice (37.3 ± 2.6 in OB » OB vs. 35.5 ± 4.48 in OB » C, *p* > 0.05). Maternal OB diet tended to increase the proportion of abnormal mitochondria within both offspring groups (i.e., in OB » C vs. C » C and in OB » OB vs. C » OB (*p* < 0.1). The percentage of oocyte abnormal mitochondria was positively correlated with offspring body weight (r = 0.613, *p* < 0.05).

When looking at the different categories of abnormal mitochondrial ultrastructure separately, we noticed that the proportions of dumbbell shaped mitochondria and degenerative mitochondria were increased by offspring diet (*p* < 0.05) whereas the proportions of elongated mitochondria, mitochondria with electron dense foci and degenerative mitochondria were increased by the maternal OB background (*p* < 0.05). The maternal effect on the proportion of mitochondria with electron dense foci was dependent on offspring diet (interaction *p* < 0.05).

Detailed information about the different categories of aberrant mitochondrial ultrastructure with the corresponding *p*-values is shown in Table 2. The separate effects of maternal and offspring diet in the pairwise comparisons are shown in Supplementary File S2.

**TABLE 2:** Mitochondrial (MT) abnormal ultrastructure in oocytes collected from C- or OB-fed offspring and born to mothers that were either fed a C or OB diet, in a 2 × 2 factorial design, categorized in elongated, rose petal shaped, e-dense and dumbbell shaped mitochondria, together with mitochondria showing lose inner membranes and mitochondrial degeneration. Data are presented as mean proportions ±S.E.M. and are derived from 1 oocyte per offspring per group, from 8 litters or mothers. *p-*values of the binary logistic regression are stated (F_0_ = maternal diet effect, F_1_ = offspring diet effect, I = interaction). Pairwise comparisons within treatment groups are presented in Supplementary File S2.

Group	Total abnormal MT	Elongated MT	Rose petal shaped MT	MT with E-dense foci	Dumpbell shaped MT	MT degeneration	MT with loose inner membranes
C » C	25.6 ± 2.16	2.0 ± 0.96	5.5 ± 1.10	2.1 ± 0.59	1.9 ± 0.70	7.3 ± 0.92	5.9 ± 1.27
C » OB	32.1 ± 1.57	2.8 ± 0.84	5.5 ± 1.04	4.0 ± 0.91	3.0 ± 0.93	9.2 ± 1.5	6.6 ± 0.95
OB » C	35.5 ± 4.48	3.4 ± 0.71	6.1 ± 1.46	5.2 ± 1.83	3.3 ± 0.90	10.9 ± 2.27	5.7 ± 2.00
OB » OB	37.3 ± 2.58	3.3 ± 0.74	4.8 ± 0.86	3.7 ± 0.79	4.0 ± 1.65	13.8 ± 1.99	6.0 ± 1.02
F_0_ *p*-value	0.001	0.005	0.715	0.042	0.115	0.000	0.674
F_1_ *p*-value	0.001	0.391	0.597	0.717	0.042	0.004	0.684
I *p*-value	0.008	0.627	0.078	0.005	0.135	0.672	0.586

### Expression of ETC complex markers in offspring oocytes

**FIGURE 8:**
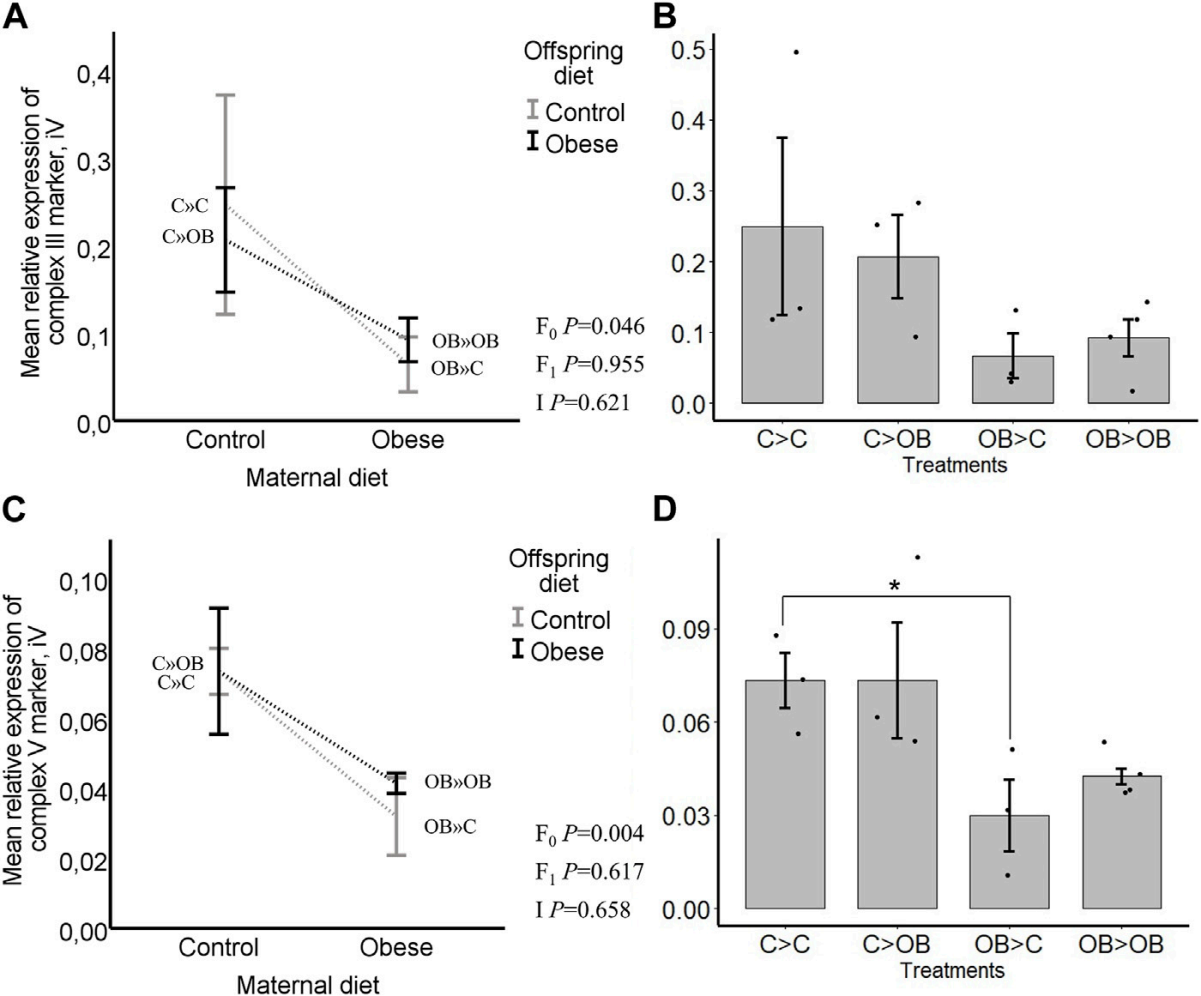
Maternal and offspring diet effects on the expression of the ETC, complexes III and V markers in oocytes. Graphs showing expression of ETC complexes III markers in oocytes collected from C- or OB-fed offspring and born to mothers that were either fed a C or OB diet, in a 2 × 2 factorial design, using an interaction plot **(A)** and a dot plot **(B)**. Graphs showing expression of ETC complexes V markers in oocytes collected from C- or OB-fed offspring and born to mothers that were either fed a C or OB diet, in a 2 × 2 factorial design, using an interaction plot **(C)** and a dot plot **(D)**. Data are presented as mean ± S.E.M. and are derived from pools of 40–80 oocytes per group, from 3 to 4 C and OB mothers. *p-*values of the main effects are stated (F_0_ = maternal diet effect, F_1_ = offspring diet effect, I = interaction). Significant differences are indicated with an asterisk (*), tendencies with a dollar sign ($). Pairwise comparisons within treatment groups are presented in Supplementary File S2.

Complex I was not detected. Offspring diet did not influence the expression of any of the OXPHOS complexes in the offspring oocytes (*p* > 0.05). Maternal diet significantly reduced the expression of complex III and complex V marker, regardless of the offspring diet (*p* < 0.05, Figure 8). Complex II and IV were not affected by a maternal OB diet and maternal x offspring diet interaction was not significant (*p* > 0.05).

Pairwise comparison within maternal diet groups showed no differences in expression of ETC, markers caused by the offspring diet. In pairwise comparisons within each offspring diet group, no differences were seen in the expression of complex III marker (in C » C, 0.25 ± 0.13 compared to OB » C, 0.06 ± 0.03; and in C » OB, 0.21 ± 0.03 compared to OB » OB 0.09 ± 0.02, Integral volume (iV) (*p* > 0.05)) but the expression of complex V marker was significantly higher in C » C (0.07 ± 0.01, iV) compared to OB » C (0.03 ± 0.01, iV, *p* < 0.05). It did not differ in C » OB (0.07 ± 0.02, iV) compared to OB » OB (0.04 ± 0.00, iV, *p* > 0.05).

### Offspring oocyte metabolic activity

**FIGURE 9:**
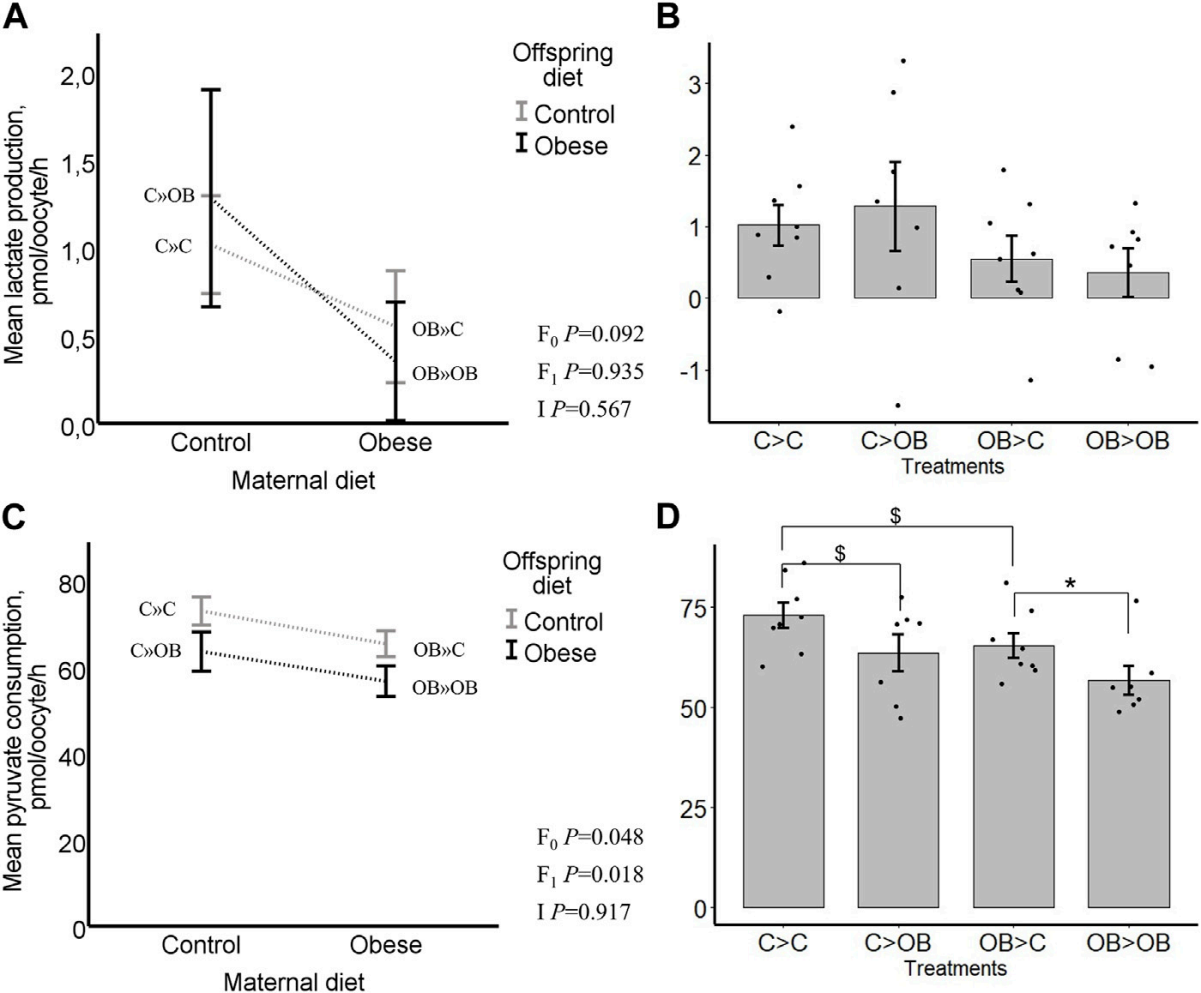
Maternal and offspring diet effects on offspring oocyte lactate production and pyruvate consumption. Graphs representing lactate production in oocytes collected from C- or OB-fed offspring and born to mothers that were either fed a C or OB diet, in a 2 × 2 factorial design using an interaction plot **(A)** and a dot plot **(B)**. Graphs representing pyruvate consumption in oocytes collected from C- or OB-fed offspring and born to mothers that were either fed a C or OB diet, in a 2 × 2 factorial design using an interaction plot **(C)** and a dot plot **(D)**. Data are presented as mean ± S.E.M. and are derived from 6-8 oocytes per offspring per group, from 8 litters or mothers. *p*-values of the main effects are stated (F_0_ = maternal diet effect, F_1_ = offspring diet effect, I = interaction). Significant differences are indicated with an asterisk (*), tendencies with a dollar sign ($). Pairwise comparisons within treatment groups are presented in Supplementary File S2.

Oocyte pyruvate consumption was significantly reduced both by offspring and by maternal OB diets (*p* < 0.05). The maternal x offspring diet interaction was not significant (Figure 9, *p* > 0.05). In pairwise comparison within each maternal diet group, OB-fed offspring had a reduced pyruvate consumption when born to OB mothers (OB » OB, 57 ± 3.5 vs. OB » C, 65 ± 3.0, pmol/oocyte/h, *p* < 0.05) whereas the pyruvate consumption only tended to differ in offspring born to C mothers (C » OB, 64 ± 4.5 vs. C » C, 73 ± 3.2, pmol/oocyte/h, *p* < 0.1). The pyruvate consumption tended to be reduced in OB » C compared to C » C (*p* < 0.1). No differences were seen between OB » OB and C » OB. The oocyte pyruvate consumption was negatively correlated with offspring body weight (r = −0.449, *p* < 0.05).

Oocyte lactate production was not affected by offspring diet (*p* > 0.05) and tended to be reduced by a maternal metabolic background (C » C, 1.02 ± 0.23; C » OB, 1.29 ± 0.61; OB » C, 0.55 ± 0.32; OB » OB, 0.35 ± 0.34, pmol/oocyte/h, *p* < 0.1). The maternal x offspring diet interaction was not significant (Figure 9, *p* > 0.05). Oocyte lactate production was not different between groups in pairwise comparisons within both maternal and offspring groups. Oocyte lactate production was not correlated with offspring body weight (r = −0.067, *p* < 0.05).

## Discussion

The aim of this study was to investigate the influence of a maternal OB background on offspring oocyte quality, when the offspring is fed either a control or an OB diet. A validated and robust outbred Swiss mouse model was used to increase the pathophysiological relevance and translatability to the human situation. We have previously shown that Swiss mice are sensitive to an obesogenic diet, develop hypercholesterolemia, obesity, and insulin resistance, reflecting in a reduced oocyte quality with mitochondrial functional and morphological alterations (Marei et al., 2020; Smits et al., 2021; Smits et al., 2022a; Moorkens et al., 2022). We focused on several complementary oocyte mitochondrial qualitative and quantitative parameters. We found that the majority of the assessed mitochondrial functions in oocytes were significantly affected by the offspring OB diet. However, the extent of this impact, at least for some outcome parameters, seemed to depend on the metabolic background of the mother. The mitochondrial changes in offspring oocytes associated with a maternal OB diet only were significant but relatively limited.

Most studies investigating the effect of diet-induced obesity on oocyte quality and fertility do not consider the potential interacting effects of the maternal metabolic background. In human, obese daughters are often born to obese mothers, making such interaction a very important factor to consider. The data generated in the present study in offspring born to control mothers are comparable with other studies using mice born to control healthy mothers, following standard breeding protocols. In these mice, we found that feeding a high fat and high sugar diet (in C » OB) significantly increased live body weight trajectory, total serum cholesterol, as well as oocyte LDC, MMP, ROS and mitochondrial ultrastructural abnormalities, compared to the control group (C » C). This is in line with previous findings from our laboratory using the same outbred Swiss model (Marei et al., 2020; Smits et al., 2021; Smits et al., 2022a), and also in line with other studies where the inbred C57BL/6 mouse model was used (Igosheva et al., 2010; Boots et al., 2016). On top, we showed that the offspring diet effect was already present at 1 week post-weaning and was influenced by the maternal metabolic background at week 7 post-weaning. In our study, offspring’s oocyte pyruvate consumption was reduced, and the mitochondrial activity and percentage of mitochondrial ultrastructural abnormalities were increased when offspring gained more weight. Consumption of an OB diet and development of obesity are known to alter the biochemical composition of the blood and follicular fluid (Leroy et al., 2022), leading to increased lipid accumulation in oocytes, which creates a nutrient overload and metabolic dysregulation resulting in lipotoxicity, oxidative stress, and mitochondrial dysfunction (Boudoures et al., 2017a; Zhao et al., 2017; Bradley and Swann, 2019; Marei et al., 2020; Smits et al., 2021; Leroy et al., 2022). We could also detect a tendency towards a reduced oocyte pyruvate consumption in C » OB compared with C » C mice. These results are thus in agreement with the expected increase in fatty acid beta-oxidation, and may explain, at least in part, the increased MMP and ROS accumulation in the affected oocytes.

While the direct impact of OB diet was confirmed in our experimental model, our main focus was to determine if the maternal OB background might influence the offspring oocyte quality and its mitochondrial functions and how such maternal impact may affect the effects of the offspring OB diet on oocyte mitochondrial function. For this, a 2 × 2 factorial design was used. The most prominent effect of maternal diet observed here was on offspring oocyte mitochondrial morphology. Maternal OB diet significantly increased the proportions of total mitochondrial ultrastructural abnormalities in offspring oocytes, and more specifically on the proportion of mitochondria classified as degenerated, elongated or electron dense. These abnormalities were limited to only 5%–10% of the mitochondria in total. Similar results have been previously reported by a few studies using C57BL/6 mice (Saben et al., 2016; Boudoures et al., 2017a), where oocyte mitochondria were found to be significantly larger and less round in offspring born to obese mothers compared to those born to lean mothers (Saben et al., 2016). The authors did not report the exact proportion of the abnormal mitochondria in the affected oocytes. Mitochondrial morphology can be linked to its function. Increased mitochondrial elongation may be a consequence of altered fusion and fission machinery due to high levels of cellular stress (Runkel et al., 2014) and may imply alterations in energy metabolism pathways (Boudoures et al., 2016; Saben et al., 2016). Increased mitochondrial electron density is linked with altered glucose and oxygen metabolism (Grindler and Moley, 2013). In our study, we detected both features being more prevalent in oocytes from OB-born offspring, together with a reduction in pyruvate consumption, suggesting maternally induced alterations in offspring oocyte energy efficiency mechanisms. However, the proportions of elongated or electron dense mitochondria were relatively low (about 2%) and only increased by 1%–2% in response to maternal and/or offspring OB diet, suggesting that the overall impact may be rather limited. The interaction between maternal diet and offspring diet effects on mitochondrial ultrastructural morphology was also significant, meaning that the effect of an offspring OB diet on offspring mitochondrial morphology is dependent on the maternal metabolic background.

It is important to keep in mind that the increased oocyte mitochondrial ultrastructural abnormalities in response to maternal and/or offspring OB diet does not necessarily mean that other mitochondrial or cellular metabolic functions are affected. Oocytes contain around hundred thousand mitochondria and display several adaptive mechanisms that may be used to compensate for energy deficit or overload (Runkel et al., 2014; Marei and Leroy, 2022). Previous studies from our laboratory have shown that the percentage of mitochondria with abnormal morphology in control Swiss oocytes is around 20% (Smits et al., 2022a). An increase of 5%–10% of mitochondrial ultrastructural abnormalities detected here may have no, or limited, functional impact. For a more comprehensive assessment, we investigated several other mitochondrial functions and indeed we found that MMP and intracellular levels of ROS were not affected by the maternal diet effects despite the observed effects on mitochondrial morphology. Assessment of MMP is commonly used as an indicator of oocyte mitochondrial activity (Blerkom et al., 2003). The MMP increases during the final stage of maturation in murine oocytes and is linked to an increase in oxidative phosphorylation and ATP production (Blerkom et al., 2003; Al-Zubaidi et al., 2019) and correlates with preimplantation development (Komatsu et al., 2014). Increased ROS concentrations in oocytes is also frequently used in other studies to indicate oxidative stress. Increased MMP and ROS in oocytes have both been linked with reduced oocyte quality, reduced developmental competence (Marei et al., 2019) and lower pregnancy rates in diet-induced obese mice (Smits et al., 2021). Interestingly, also in C57BL/6 mice, maternal diet-induced obesity did not influence ATP production in the oocytes of the offspring despite the associated increase in mitochondrial ultrastructural abnormalities (Boudoures et al., 2017b). Offspring in the later study were fed a normal chow diet. Here we also found no maternal effects on oocyte MMP and ROS even in offspring fed an OB diet, showing that the maternal metabolic background did not increase the sensitivity of the oocyte to a high fat and high sugar diet. It is also interesting to mention that among the different classes of abnormal oocyte mitochondria in offspring from OB mothers, degenerated mitochondria were the most prevalent. This might be a sign of clearance of damaged mitochondria rather than retaining the defective ones. We suggest that, as oocytes do not have the capacity to efficiently remove damaged mitochondria (Boudoures et al., 2017c), they eventually degenerate.

It was also important in the present study to examine the effects on mitochondrial DNA copy numbers. The results were highly variable within each treatment group and we could not detect any significant effect of maternal or offspring diet. There was a numerical decrease in mitochondrial DNA copy numbers in C » OB oocytes compared to C » C, which is in line with our previous report (Marei et al., 2020). Our data are in contrast with the data reported using the inbred C57BL6 mice were maternal obesity resulted in a significant reduction in mitochondrial DNA copy numbers in the offspring’s oocytes (Saben et al., 2016). Mitochondrial DNA copy numbers are tightly regulated by the rate of mitochondrial biogenesis and replication, as well as the rate of mitophagy by which damaged mitochondria might be removed. Mitochondrial biogenesis is active in primordial germ cells and during early stages of folliculogenesis (St John, 2019). High levels of cellular metabolic stress can influence mitochondrial regulation of fusion and fission and alter mitochondrial DNA copy numbers (Fukunaga, 2021). Therefore, if the maternal metabolic effects on oocyte mitochondrial functions would persist during prenatal and postnatal development, or if mitochondrial dysfunction would be transmitted through the female germline as suggested in inbred mice, we would expect an impact on mitochondrial DNA copy numbers and mitochondrial bioenergetic functions in the offspring oocytes. In the present study using outbred Swiss mice, we were unable to detected such impact.

Oocytes normally contain a heterogenous population of active and inactive mitochondria (Van Blerkom, 2011). Active mitochondria move towards the peri-cortical area of the oocyte during the final phase of oocyte maturation to support the cortical granule reaction to prevent polyspermy and provide energy for polar body extrusion (Kirillova et al., 2021). A peri-cortical distribution of active mitochondria also guarantees an equal division of the mitochondria among the blastomeres after cleavage and is therefore of the utmost importance for optimal embryo development (Liu et al., 2003; Van Blerkom and Davis, 2006; 2007; Van Blerkom, 2008). In our study, the mitochondrial distribution was not affected by offspring and maternal diets. We did however observe aberrant mitochondrial uncoupling and clustering in a few oocytes only in offspring born to obese mothers. Mitochondrial clustering may imply alterations in oocyte energy production due to overexpression of mitochondrial fusion proteins (Babayev and Seli, 2015). Igosheva et al. (2010) and Hou et al. (2016) reported clusters of mitochondria in some oocytes of obese mice being indicative of metabolic or functional damage, involving a greater need for energy production to preserve oocyte viability and competence. Nevertheless, since these features were only limited to 6%–8% of the oocytes, with a normal MMP and active mitochondrial distribution in all the rest, the potential impact may also be limited. In addition, not only clustering but also mitochondrial lactate and pyruvate serve as a redox buffer that equilibrates the NADH/NAD^+^ ratio across the cell. Lactate acts, amongst other functions, as an important redox carrier. Its production is increased in anaerobic respiration when glucose is converted into lactic acid, being energetically less efficient than oxidative phosphorylation (Lagarde et al., 2021). We could not detect differences in offspring oocyte lactate production between treatment groups. However, both offspring and maternal OB diet reduced the consumption of pyruvate, the principle energy substrate of the oocyte during final maturation (Richani et al., 2021) with the lowest pyruvate consumption seen in OB-born OB fed offspring. Oocytes with reduced pyruvate may be more sensitive towards increased ROS production, as a reduced pyruvate consumption may lead to reduced glutathione levels in oocytes (Funahashi et al., 2008). We could indeed detect dietary induced increased ROS accumulation in the oocyte, but only due to offspring OB diet and not linked with the mother’s OB background. The reduced pyruvate consumption together with the numerical reduction in lactate production may suggest a change in the metabolic preference towards β-oxidation for energy provision. However, no differences could be detected in oocyte LDC, MMP or ROS to support this notion.

Finally, this study is the first to investigate the potential effect of maternal and offspring OB diet on the expression of protein complexes of the electron transport chain in oocytes. Complex I was not detected, which is line with very recent observations reported by Rodriguez-Nuevo et al. (2022), who illustrated that supressing complex I in oocytes is an intrinsic mechanisms to maintain ROS-free mitochondrial metabolism. In the present study, this appears not to be changed by maternal or offspring diet. Also no differences in expression of complex II and IV markers were detected. Complex I and II oxidize NADH and FADH_2_ respectively, transferring the resulting electrons to ubiquinol, which carries electrons to complex III to be subsequently used for ATP production (Rutter et al., 2010). In the absence of complex I, complex II activity becomes crucial for the function of the ETC and for energy production but it was not influenced by diets. We also found that the expression of the complex III and V markers were significantly reduced by maternal OB diet. However, in the pairwise comparisons, this was only confirmed for complex V and only in offspring fed a control diet. There was a tendency for a lower complex III expression in OB fed offspring born to OB mothers. Complex III uses energy released in downhill electron transfers to pump more protons across the inner mitochondrial membrane (Chandel, 2010). The resulting proton gradient (MMP) is then used to produce ATP by the fifth complex, also known as ATP-synthase (Lenaz et al., 2006; Zhao et al., 2019; Maclean et al., 2022). Reactive oxygen species are produced as a by-product of the ETC (Brand, 2010). Protective mechanisms by reducing expression of ROS generating ETC, complexes have been reported by others in somatic tissue (Gleason et al., 2011; Le Vasseur et al., 2021; Groen et al., 2022). Therefore, the slightly reduced expression of complex III in OB » OB oocytes may be a beneficial compensatory mechanism for the oocytes to control ROS production. Since MMP was not affected, no consequence for energy production is expected. Lower complex V expression in OB » C oocytes compared to C » C is very interesting and may be secondary to metabolic adaptations also leading to reduced pyruvate consumption and suggesting a lower metabolic activity in these oocytes. However, these adaptations were not sufficient to impact MMP.

It is also important to highlight that, except for specific mitochondrial ultrastructural features, there was no interaction between the maternal diet and offspring diet effects. Following the systematic 2 × 2 factorial design of the data, we did not directly compare OB » OB oocyte quality measures with those of the C » C. Nevertheless, we can clearly see from the figures that the highest degree of alteration or change in most outcome parameters were due to the combined effect of maternal and offspring OB diets. The specific maternal contribution of these differences in some parameters are evident but not significant. In our study, oocytes were collected of 10 weeks old offspring. It is possible that treatment effects will become more pronounced when offspring age increases, especially since aging is linked with mitochondrial damage (Sun et al., 2016). Therefore the functional impact of the maternal OB background on offspring oocyte mitochondria may still become significant over time. Also, our experimental design tried to mimic the societal situation in which daughters may be raised in the same OB environment after weaning. Therefore, the present study does not allow to determine the specific window of impact during which the maternal diet could have resulted in reduced quality of the daughter’s oocyte. However, our data do suggest that the pool of dormant oocytes in the ovary of the foetus or the newly born pup is already sensitive to an adverse metabolic condition leading to mitochondrial defects in the offspring oocytes at adult age, even if the post-weaning diet was perfectly healthy. Similar long-term carry over effects on oocyte mitochondrial features were reported also in our previous study in obese mice submitted to diet normalisation for 4 weeks (Smits et al., 2022b). In our study, litter sizes were reduced to allow equal milk supply per pup. However, litter size is linked with offspring body weight and the expression of each phenotype as such may be relevant to fully understand maternal-offspring nutrient interactions in developmental studies. Also, artificial reduction of litter size itself can be considered as a confounding factor, as it may affect maternal care behaviour and influence offspring health (Enes-Marques and Giusti-Paiva, 2018; Briffa et al., 2019; Xavier et al., 2019). Nevertheless, our data show that litter size at birth was similar between C- and OB-fed mothers and hence did not act as a confounding factor in this study. Finally, maternal obese diets during pregnancy and lactation may redirect metabolic programming resulting in an altered offspring metabolism (Wells, 2014; Gawlinska et al., 2021; Furse et al., 2022). This may indirectly affect oocyte quality in the offspring.

This study is the first to use an outbred model to investigate the effect of both maternal and offspring OB diets and their interaction on offspring oocyte mitochondrial ultrastructure and function. We show evidence that especially the offspring diet had the most obvious impact on mitochondrial features and thus, the most obvious impact on oocyte quality. However, the extent of this impact was often dependent on the maternal metabolic background with the most prominent effects seen in oocytes from OB offspring born to OB mothers. The maternal OB background on its own appears to have a limited but significant impact on the offspring’s oocyte mitochondrial morphology but not on bioenergetic functions, on mitochondrial DNA copy numbers, mitochondrial activity (estimated by MMP), distribution of active mitochondria, as well as on ROS levels. These results strongly suggest that aberrant mitochondria in oocytes (induced by OB diet) are not transferred through the female germline to the next-generation. The majority of abnormal oocyte mitochondria in OB-born offspring were degenerated suggesting that, at least in this outbred model, defective mitochondria can be removed and replaced without compromising mitochondrial DNA copy numbers. Reduced oocyte pyruvate consumption and lower expression of the ETC complex V marker without a significant change in MMP, ROS and lactate production indicates that these adaptations may have no, or limited, functional consequences. These results are in contrast with previous reports using inbred models and highlight marked differences in responses between inbred and outbred mice strains. Therefore, extrapolation of data from inbred mouse models, particularly related to oocyte mitochondrial functions and intergenerational effects of metabolic stress, should be done with caution. Pathophysiologically relevant outbred mouse models should be used in further studies to investigate the potential effects on somatic cell functions in the offspring born to obese mothers, the developmental competence of their oocytes and potential effects on the second generation.

## Data Availability

The datasets presented in this study can be found in online repositories. The names of the repository/repositories and accession number(s) can be found below: https://data.mendeley.com/drafts/hc446bk7bd/1, The Impact of a Maternal and Offspring Obesogenic diet on Daughter’s Oocyte Mitochondrial Ultrastructure and Bioenergetic Responses. Insights from an Outbred Mouse Model.

## Abbreviations

OB, obesogenic; C, control; C»C, offspring born to a control mother and fed a control diet; C»OB, offspring born to a obese mother and fed an obese mother; OB»C, offspring born to an obese mother and fed a control diet; OB»OB, offspring born to an obese mother and fed an obesogenic diet; F_0_, maternal; F_1_, offspring; I, interaction; COC, cumulus oocyte complex; LDC, lipid droplet content; MMP, mitochondrial inner membrane potential; ROS, reactive oxygen species; OXPHOS, oxidative phosphorylation; ETC, electron transport chain; iV, integral volume.

## References

[B1] Al-Zubaidi U. Liu J. Cinar O. Robker R. L. Adhikari D. Carroll J. (2019). The spatio-temporal dynamics of mitochondrial membrane potential during oocyte maturation. Mol. Hum. Reprod. 25 (11), 695–705. 10.1093/molehr/gaz055 31579926 PMC6884418

[B2] Allain C. C. Poon L. S. Chan C. S. G. Richmond W. Fu P. C. (1974). Enzymatic determination of total serum-cholesterol. Clin. Chem. 20 (4), 470–475. 10.1093/clinchem/20.4.470 4818200

[B3] Andreas E. Reid M. Zhang W. Moley K. H. (2019). The effect of maternal high-fat/high-sugar diet on offspring oocytes and early embryo development. Mol. Hum. Reprod. 25 (11), 717–728. 10.1093/molehr/gaz049 31588490 PMC6884416

[B4] Antunes D. M. F. Kalmbach K. K. Wang F. Seth-Smith M. L. Kramer Y. Kohlrausch F. B. (2014). Oocytes from women with diminished ovarian reserve and obesity have shortened telomeres. Fertil. Steril. 102(3), E331–E331. DOI 10.1016/j.fertnstert.2014.07.1120

[B5] Armitage J. A. Poston L. Taylor P. D. (2008). Developmental origins of obesity and the metabolic syndrome: the role of maternal obesity. Front. Horm. Res. 36, 73–84. 10.1159/000115355 18230895

[B6] Babayev E. Seli E. (2015). Oocyte mitochondrial function and reproduction. Curr. Opin. Obstet. Gynecol. 27 (3), 175–181. 10.1097/GCO.0000000000000164 25719756 PMC4590773

[B7] Bansal A. Simmons R. A. (2018). Epigenetics and developmental origins of diabetes: correlation or causation? Am. J. Physiol. Endocrinol. Metab. 315 (1), E15–E28. 10.1152/ajpendo.00424.2017 29406781 PMC6334998

[B8] Blerkom J. V. Davis P. Alexander S. (2003). Inner mitochondrial membrane potential (DeltaPsim), cytoplasmic ATP content and free Ca2+ levels in metaphase II mouse oocytes. Hum. Reprod. 18 (11), 2429–2440. 10.1093/humrep/deg466 14585897

[B9] Blondeau B. Joly B. Perret C. Prince S. Bruneval P. Lelievre-Pegorier M. (2011). Exposure *in utero* to maternal diabetes leads to glucose intolerance and high blood pressure with no major effects on lipid metabolism. Diabetes Metab. 37 (3), 245–251. 10.1016/j.diabet.2010.10.008 21257329

[B10] Boots C. E. Boudoures A. Zhang W. Drury A. Moley K. H. (2016). Obesity-induced oocyte mitochondrial defects are partially prevented and rescued by supplementation with co-enzyme Q10 in a mouse model. Hum. Reprod. 31 (9), 2090–2097. 10.1093/humrep/dew181 27432748 PMC4991662

[B11] Boudoures A. L. Chi M. Thompson A. Zhang W. Moley K. H. (2016). The effects of voluntary exercise on oocyte quality in a diet-induced obese murine model. Reproduction 151 (3), 261–270. 10.1530/REP-15-0419 26700938 PMC4821597

[B12] Boudoures A. L. Saben J. Drury A. Scheaffer S. Modi Z. Zhang W. (2017a). Obesity-exposed oocytes accumulate and transmit damaged mitochondria due to an inability to activate mitophagy. Dev. Biol. 426 (1), 126–138. 10.1016/j.ydbio.2017.04.005 28438607

[B13] Boudoures A. L. Saben J. Drury A. Scheaffer S. Modi Z. Zhang W. (2017b). Obesity-exposed oocytes accumulate and transmit damaged mitochondria due to an inability to activate mitophagy. Dev. Biol. 426, 126–138. 10.1016/j.ydbio.2017.04.005 28438607

[B14] Boudoures A. L. Saben J. Drury A. Scheaffer S. Modi Z. Zhang W. (2017c). Obesity-exposed oocytes accumulate and transmit damaged mitochondria due to an inability to activate mitophagy. Dev. Biol. 426 (1), 126–138. 10.1016/j.ydbio.2017.04.005 28438607

[B15] Bradley J. Swann K. (2019). Mitochondria and lipid metabolism in mammalian oocytes and early embryos. Int. J. Dev. Biol. 63 (3-4-5), 93–103. 10.1387/ijdb.180355ks 31058306

[B16] Brand M. D. (2010). The sites and topology of mitochondrial superoxide production. Exp. Gerontol. 45 (7-8), 466–472. 10.1016/j.exger.2010.01.003 20064600 PMC2879443

[B17] Briffa J. F. O'Dowd R. Romano T. Muhlhausler B. S. Moritz K. M. Wlodek M. E. (2019). Reducing pup litter size alters early postnatal calcium homeostasis and programs adverse adult cardiovascular and bone health in male rats. Nutrients 11 (1), 118. ARTN. 10.3390/nu11010118 30626125 PMC6356436

[B18] Broughton D. E. Moley K. H. (2017). Obesity and female infertility: potential mediators of obesity's impact. Fertil. Steril. 107 (4), 840–847. 10.1016/j.fertnstert.2017.01.017 28292619

[B19] Cardozo E. Pavone M. E. Hirshfeld-Cytron J. E. (2011). Metabolic syndrome and oocyte quality. Trends Endocrinol. Metab. 22 (3), 103–109. 10.1016/j.tem.2010.12.002 21277789

[B20] Chandel N. S. (2010). Mitochondrial complex III: an essential component of universal oxygen sensing machinery? Respir. Physiology Neurobiol. 174 (3), 175–181. 10.1016/j.resp.2010.08.004 PMC2991558 20708106

[B21] Cox R. T. Poulton J. Williams S. A. (2021). The role of mitophagy during oocyte aging in human, mouse, and Drosophila: implications for oocyte quality and mitochondrial disease. Reproduction Fertil. 2 (4), R113–R129. 10.1530/Raf-21-0060 PMC8801022 35118415

[B22] De Biasi S. Gibellini L. Cossarizza A. (2015). Uncompensated polychromatic analysis of mitochondrial membrane potential using JC-1 and multilaser excitation. Curr. Protoc. Cytom. 72, 31–732. 10.1002/0471142956.cy0732s72 25827483

[B23] Dearden L. Ozanne S. E. (2015). Early life origins of metabolic disease: developmental programming of hypothalamic pathways controlling energy homeostasis. Front. Neuroendocrinol. 39, 3–16. 10.1016/j.yfrne.2015.08.001 26296796

[B24] Desmet K. L. J. Van Hoeck V. Gagne D. Fournier E. Thakur A. O'Doherty A. M. (2016). Exposure of bovine oocytes and embryos to elevated non-esterified fatty acid concentrations: integration of epigenetic and transcriptomic signatures in resultant blastocysts. Bmc Genomics 17, 1004. 10.1186/s12864-016-3366-y 27931182 PMC5146907

[B25] Enes-Marques S. Giusti-Paiva A. (2018). Litter size reduction accentuates maternal care and alters behavioral and physiological phenotypes in rat adult offspring. J. Physiological Sci. 68 (6), 789–798. 10.1007/s12576-018-0594-8 PMC10717135 29380149

[B26] Engin A. B. (2017). What is lipotoxicity? Obes. Lipotoxicity 960, 197–220. 10.1007/978-3-319-48382-5_8 28585200

[B27] Fukunaga H. (2021). Mitochondrial DNA copy number and developmental origins of health and disease (DOHaD). Int. J. Mol. Sci. 22 (12), 6634. 10.3390/ijms22126634 34205712 PMC8235559

[B28] Funahashi H. Koike T. Sakai R. (2008). Effect of glucose and pyruvate on nuclear and cytoplasmic maturation of porcine oocytes in a chemically defined medium. Theriogenology 70 (7), 1041–1047. 10.1016/j.theriogenology.2008.06.025 18657854

[B29] Furse S. Koulman A. Ozanne S. E. Poston L. White S. L. Meek C. L. (2022). Altered lipid metabolism in obese women with gestational diabetes and associations with offspring adiposity. J. Clin. Endocrinol. Metabolism 107 (7), E2825–E2832. 10.1210/clinem/dgac206 PMC9757862 35359001

[B30] Gahagan S. Uauy R. Roseboom T. J. (2012). Developmental origins of pediatric obesity. Int. J. Pediatr. 2012, 309863. 10.1155/2012/309863 22934119 PMC3426220

[B31] Gaillard R. (2015). Maternal obesity during pregnancy and cardiovascular development and disease in the offspring. Eur. J. Epidemiol. 30 (11), 1141–1152. 10.1007/s10654-015-0085-7 26377700 PMC4684830

[B32] Gardner D. K. Lane M. (2013). “Assessment of nutrient uptake, metabolic activity,” in A laboratory guide to the mammalian embryo (Oxford): O.U. Press.), 139–153.

[B33] Gawlinska K. Gawlinski D. Filip M. Przegalinski E. (2021). Relationship of maternal high-fat diet during pregnancy and lactation to offspring health. Nutr. Rev. 79 (6), 709–725. 10.1093/nutrit/nuaa020 32447401

[B34] Gleason C. Huang S. B. Thatcher L. F. Foley R. C. Anderson C. R. Carroll A. J. (2011). Mitochondrial complex II has a key role in mitochondrial-derived reactive oxygen species influence on plant stress gene regulation and defense. Proc. Natl. Acad. Sci. U. S. A. 108 (26), 10768–10773. 10.1073/pnas.1016060108 21670306 PMC3127871

[B35] Gonzalez M. B. Robker R. L. Rose R. D. (2022). Obesity and oocyte quality: significant implications for ART and emerging mechanistic insights. Biol. Reprod. 106 (2), 338–350. 10.1093/biolre/ioab228 34918035

[B36] Grindler N. M. Moley K. H. (2013). Maternal obesity, infertility and mitochondrial dysfunction: potential mechanisms emerging from mouse model systems. Mol. Hum. Reprod. 19 (8), 486–494. 10.1093/molehr/gat026 23612738 PMC3712655

[B37] Groen C. M. Podratz J. L. Pathoulas J. Staff N. Windebank A. J. (2022). Genetic reduction of mitochondria complex I subunits is protective against cisplatin-induced neurotoxicity in Drosophila. J. Neurosci. 42 (5), 922–937. 10.1523/Jneurosci.1479-20.2021 34893548 PMC8808726

[B38] Hou Y. J. Zhu C. C. Duan X. Liu H. L. Wang Q. Sun S. C. (2016). Both diet and gene mutation induced obesity affect oocyte quality in mice. Sci. Rep. 6, 18858. 10.1038/srep18858 26732298 PMC4702149

[B39] Igosheva N. Abramov A. Y. Poston L. Eckert J. J. Fleming T. P. Duchen M. R. (2010). Maternal diet-induced obesity alters mitochondrial activity and redox status in mouse oocytes and zygotes. PLoS One 5 (4), e10074. 10.1371/journal.pone.0010074 20404917 PMC2852405

[B40] Jialal I. Adams-Huet B. Duong F. Smith G. (2014). Relationship between retinol-binding protein-4/adiponectin and leptin/adiponectin ratios with insulin resistance and inflammation. Metab. Syndr. Relat. Disord. 12 (4), 227–230. 10.1089/met.2014.0013 24593134

[B41] Johns D. J. Lindroos A. K. Jebb S. A. Sjostrom L. Carlsson L. M. Ambrosini G. L. (2015). Dietary patterns, cardiometabolic risk factors, and the incidence of cardiovascular disease in severe obesity. Obes. (Silver Spring) 23 (5), 1063–1070. 10.1002/oby.20920 PMC6680188 25865622

[B42] Jungheim E. S. Schoeller E. L. Marquard K. L. Louden E. D. Schaffer J. E. Moley K. H. (2010). Diet-induced obesity model: abnormal oocytes and persistent growth abnormalities in the offspring. Endocrinology 151 (8), 4039–4046. 10.1210/en.2010-0098 20573727 PMC2940512

[B43] Keleher M. R. Zaidi R. Shah S. Oakley M. E. Pavlatos C. El Idrissi S. (2018). Maternal high-fat diet associated with altered gene expression, DNA methylation, and obesity risk in mouse offspring. PLoS One 13 (2), e0192606. 10.1371/journal.pone.0192606 29447215 PMC5813940

[B44] Keller K. B. Lemberg L. (2003). Obesity and the metabolic syndrome. Am. J. Crit. Care 12 (2), 167–170. 10.4037/ajcc2003.12.2.167 12625176

[B45] Kirillova A. Smitz J. E. J. Sukhikh G. T. Mazunin I. (2021). The role of mitochondria in oocyte maturation. Cells 10 (9), 2484. 10.3390/cells10092484 34572133 PMC8469615

[B46] Komatsu K. Iwase A. Mawatari M. Wang J. Yamashita M. Kikkawa F. (2014). Mitochondrial membrane potential in 2-cell stage embryos correlates with the success of preimplantation development. Reproduction 147 (5), 627–638. 10.1530/REP-13-0288 24459207

[B47] Lagarde D. Jeanson Y. Portais J. C. Galinier A. Ader I. Casteilla L. (2021). Lactate fluxes and plasticity of adipose tissues: a redox perspective. Front. Physiology 12, 689747. 10.3389/fphys.2021.689747 PMC8278056 34276410

[B48] Le Vasseur M. Friedman J. Jost M. Xu J. W. Yamada J. Kampmann M. (2021). Genome-wide CRISPRi screening identifies OCIAD1 as a prohibitin client and regulatory determinant of mitochondrial Complex III assembly in human cells. Elife 10, e67624. ARTN e6762410.7554/eLife.67624. 10.7554/eLife.67624 34034859 PMC8154037

[B49] Leary C. Leese H. J. Sturmey R. G. (2015). Human embryos from overweight and obese women display phenotypic and metabolic abnormalities. Hum. Reprod. 30 (1), 122–132. 10.1093/humrep/deu276 25391239

[B50] Lee H. S. Ma H. Juanes R. C. Tachibana M. Sparman M. Woodward J. (2012). Rapid mitochondrial DNA segregation in primate preimplantation embryos precedes somatic and germline bottleneck. Cell Rep. 1 (5), 506–515. 10.1016/j.celrep.2012.03.011 22701816 PMC3372871

[B51] Lenaz G. Fato R. Genova M. L. Bergamini C. Bianchi C. Biondi A. (2006). Mitochondrial complex I: structural and functional aspects. Biochimica Biophysica Acta-Bioenergetics 1757 (9-10), 1406–1420. 10.1016/j.bbabio.2006.05.007 16828051

[B52] Leroy J. Meulders B. Moorkens K. Xhonneux I. Slootmans J. De Keersmaeker L. (2022). Maternal metabolic health and fertility: we should not only care about but also for the oocyte. Reprod. Fertil. Dev. 35 (2), 1–18. 10.1071/RD22204 36592978

[B53] Liu M. Sims D. Calarco P. Talbot P. (2003). Biochemical heterogeneity, migration, and pre-fertilization release of mouse oocyte cortical granules. Reprod. Biol. Endocrinol. 1, 77. 10.1186/1477-7827-1-77 14613547 PMC305340

[B54] Liu T. Liu D. Song X. Qu J. Zheng X. Li J. (2021). Lipid metabolism was associated with oocyte *in vitro* maturation in women with polycystic ovarian syndrome undergoing unstimulated natural cycle. Front. Cell Dev. Biol. 9, 719173. 10.3389/fcell.2021.719173 34540838 PMC8446356

[B55] Maclean A. E. Hayward J. A. Huet D. van Dooren G. G. Sheiner L. (2022). The mystery of massive mitochondrial complexes: the apicomplexan respiratory chain. Trends Parasitol. 38 (12), 1041–1052. 10.1016/j.pt.2022.09.008 36302692 PMC10434753

[B56] Marei W. F. A. Leroy J. (2022). Cellular stress responses in oocytes: molecular changes and clinical implications. Adv. Exp. Med. Biol. 1387, 171–189. 10.1007/5584_2021_690 34921349

[B57] Marei W. F. A. Smits A. Mohey-Elsaeed O. Pintelon I. Ginneberge D. Bols P. E. J. (2020). Differential effects of high fat diet-induced obesity on oocyte mitochondrial functions in inbred and outbred mice. Sci. Rep. 10 (1), 9806. 10.1038/s41598-020-66702-6 32555236 PMC7299992

[B58] Marei W. F. A. Van den Bosch L. Pintelon I. Mohey-Elsaeed O. Bols P. E. J. Leroy J. (2019). Mitochondria-targeted therapy rescues development and quality of embryos derived from oocytes matured under oxidative stress conditions: a bovine *in vitro* model. Hum. Reprod. 34 (10), 1984–1998. 10.1093/humrep/dez161 31625574

[B59] McMurray F. MacFarlane M. Kim K. Patten D. A. Wei-LaPierre L. Fullerton M. D. (2019). Maternal diet-induced obesity alters muscle mitochondrial function in offspring without changing insulin sensitivity. FASEB J. 33 (12), 13515–13526. 10.1096/fj.201901150R 31581846

[B60] Meulders B. Marei W. F. A. Bols P. E. Leroy J. L. (2022). Effects of palmitic acid-induced lipotoxicity on epigenetic programming in zygotes and morulas during bovine *in vitro* embryo production. Anim. Reprod., e22204. 10.1071/RDv35n2Ab2 PMC10709407 38066095

[B61] Moorkens K. Leroy J. Verheyen S. Marei W. F. A. (2022). Effects of an obesogenic diet on the oviduct depend on the duration of feeding. PLoS One 17 (9), e0275379. 10.1371/journal.pone.0275379 36174086 PMC9522283

[B62] Pfledderer C. D. Gren L. H. Metos J. Brusseau T. A. O’Toole K. Buys S. S. (2021). Mothers’ diet and family income predict daughters’ healthy eating. Prev. Chronic Dis. 18, E24. 10.5888/pcd18.200445 33734964 PMC7986974

[B63] Richani D. Dunning K. R. Thompson J. G. Gilchrist R. B. (2021). Metabolic co-dependence of the oocyte and cumulus cells: essential role in determining oocyte developmental competence. Hum. Reprod. Update 27 (1), 27–47. 10.1093/humupd/dmaa043 33020823

[B64] Robker R. L. (2008). Evidence that obesity alters the quality of oocytes and embryos. Pathophysiology 15 (2), 115–121. 10.1016/j.pathophys.2008.04.004 18599275

[B65] Rodriguez-Nuevo A. Torres-Sanchez A. Duran J. M. De Guirior C. Martinez-Zamora M. A. Boke E. (2022). Oocytes maintain ROS-free mitochondrial metabolism by suppressing complex I. Nature 607(7920), 756-+. 761. 10.1038/s41586-022-04979-5 35859172 PMC9329100

[B66] Roeschla P. Bernt E. Gruber W. (1974). Enzymatic determination of total cholesterol in serum. Z. Fur Klin. Chem. Und Klin. Biochem. 12 (5), 226. 4440114

[B67] Runkel E. D. Baumeister R. Schulze E. (2014). Mitochondrial stress: balancing friend and foe. Exp. Gerontol. 56, 194–201. 10.1016/j.exger.2014.02.013 24603155

[B68] Rutter J. Winge D. R. Schiffman J. D. (2010). Succinate dehydrogenase - assembly, regulation and role in human disease. Mitochondrion 10 (4), 393–401. 10.1016/j.mito.2010.03.001 20226277 PMC2874626

[B69] Saben J. L. Boudoures A. L. Asghar Z. Thompson A. Drury A. Zhang W. (2016). Maternal metabolic syndrome programs mitochondrial dysfunction via germline changes across three generations. Cell Rep. 16 (1), 1–8. 10.1016/j.celrep.2016.05.065 27320925 PMC4957639

[B70] Samuelsson A. M. Matthews P. A. Argenton M. Christie M. R. McConnell J. M. Jansen E. H. (2008). Diet-induced obesity in female mice leads to offspring hyperphagia, adiposity, hypertension, and insulin resistance: a novel murine model of developmental programming. Hypertension 51 (2), 383–392. 10.1161/HYPERTENSIONAHA.107.101477 18086952

[B71] Smits A. Marei W. F. A. De Neubourg D. Leroy J. (2021). Diet normalization or caloric restriction as a preconception care strategy to improve metabolic health and oocyte quality in obese outbred mice. Reprod. Biol. Endocrinol. 19 (1), 166. 10.1186/s12958-021-00848-4 34736458 PMC8567997

[B72] Smits A. Marei W. F. A. Moorkens K. Bols P. E. J. De Neubourg D. Leroy J. L. M. R. (2022b). Obese outbred mice only partially benefit from diet normalization or calorie restriction as preconception care interventions to improve metabolic health and oocyte quality. Hum. Reprod. 37 (12), 2867–2884. 10.1093/humrep/deac226 36342870

[B73] Smits A. Marei W. F. A. Moorkens K. Bols P. E. J. De Neubourg D. Leroy J. (2022a). Obese outbred mice only partially benefit from diet normalization or calorie restriction as preconception care interventions to improve metabolic health and oocyte quality. Hum. Reprod. 37 (12), 2867–2884. 10.1093/humrep/deac226 36342870

[B74] St John J. C. (2019). Mitochondria and female germline stem cells-A mitochondrial DNA perspective. Cells 8 (8), 852. 10.3390/cells8080852 31398797 PMC6721711

[B75] Sun N. Youle R. J. Finkel T. (2016). The mitochondrial basis of aging. Mol. Cell 61 (5), 654–666. 10.1016/j.molcel.2016.01.028 26942670 PMC4779179

[B76] Taschereau A. Thibeault K. Allard C. Juvinao-Quintero D. Perron P. Lutz S. M. (2023). Maternal glycemia in pregnancy is longitudinally associated with blood DNAm variation at the FSD1L gene from birth to 5 years of age. Clin. Epigenetics 15 (1), 107. 10.1186/s13148-023-01524-7 37386647 PMC10308691

[B77] Van Blerkom J. Davis P. (2006). High-polarized (Delta Psi m(HIGH)) mitochondria are spatially polarized in human oocytes and early embryos in stable subplasmalemmal domains: developmental significance and the concept of vanguard mitochondria. Reprod. Biomed. Online 13, 246–254. 10.1016/s1472-6483(10)60622-0 16895640

[B78] van Blerkom J. davis P. (2007). Mitochondrial signaling and fertilization. Mol. Hum. Reprod. 13, 759–770. 10.1093/molehr/gam068 17893093

[B79] Van Blerkom J. (2008). Mitochondria as regulatory forces in oocytes, preimplantation embryos and stem cells. Reprod. Biomed. Online 16, 553–569. 10.1016/s1472-6483(10)60463-4 18413065

[B80] Van Blerkom J. (2011). Mitochondrial function in the human oocyte and embryo and their role in developmental competence. Mitochondrion 11 (5), 797–813. 10.1016/j.mito.2010.09.012 20933103

[B81] Van Hoeck V. Leroy J. L. Arias Alvarez M. Rizos D. Gutierrez-Adan A. Schnorbusch K. (2013). Oocyte developmental failure in response to elevated nonesterified fatty acid concentrations: mechanistic insights. Reproduction 145 (1), 33–44. 10.1530/REP-12-0174 23108110

[B82] Wells J. C. (2014). Adaptive variability in the duration of critical windows of plasticity: implications for the programming of obesity. Evol. Med. Public Health 2014 (1), 109–121. 10.1093/emph/eou019 25095791 PMC4148720

[B83] Wu L. L. Dunning K. R. Yang X. Russell D. L. Lane M. Norman R. J. (2010). High-fat diet causes lipotoxicity responses in cumulus-oocyte complexes and decreased fertilization rates. Endocrinology 151 (11), 5438–5445. 10.1210/en.2010-0551 20861227

[B84] Xavier J. L. P. Scomparin D. X. Pontes C. C. Ribeiro P. R. Cordeiro M. M. Marcondes J. A. (2019). Litter size reduction induces metabolic and histological adjustments in dams throughout lactation with early effects on offspring. An. Da Acad. Bras. De. Ciencias 91 (1), e20170971. 10.1590/0001-3765201920170971 30916150

[B85] Zhao L. Lu T. Gao L. Fu X. Zhu S. Hou Y. (2017). Enriched endoplasmic reticulum-mitochondria interactions result in mitochondrial dysfunction and apoptosis in oocytes from obese mice. J. Anim. Sci. Biotechnol. 8, 62. 10.1186/s40104-017-0195-z 28781772 PMC5537973

[B86] Zhao R. Z. Jiang S. Zhang L. Yu Z. B. (2019). Mitochondrial electron transport chain, ROS generation and uncoupling (Review). Int. J. Mol. Med. 44 (1), 3–15. 10.3892/ijmm.2019.4188 31115493 PMC6559295

