# Supplementary material for: The impact of a maternal and offspring obesogenic diet on daughter’s oocyte mitochondrial ultrastructure and bioenergetic responses. Insights from an outbred mouse model
Source: Front Physiol. 2023 Oct 25;14:1288472. doi: 10.3389/fphys.2023.1288472 (PMC10642210; doi:10.3389/fphys.2023.1288472)
Supplement: Supplementary file 1 [file DataSheet2.docx]

**Manuscript title: The Impact of a Maternal and Offspring Obesogenic diet on Daughter’s Oocyte Mitochondrial Ultrastructure and Bioenergetic Responses. Insights from an Outbred Mouse Model.**

**Supplementary Information 2: Pairwise comparisons**

| Table S2: Overview of all investigated outcome parameters with main effects and pairwise comparisons within treatment groups. Significant differences are indicated by an asterisk (*), while tendencies are indicated by a dollar sign ($). NS, not-significant; ND, not-detected. Arrows show the direction of change in each comparison. | | | | | | | |
| --- | --- | --- | --- | --- | --- | --- | --- |
|  | Main effects | | | In C fed offspring only | In OB fed  offspring only | In offspring from C fed mothers only | In offspring from OB fed  mothers only |
| Parameter | Maternal diet effect | Offspring diet effect | Inter-action | Maternal diet effect | Maternal diet effect | Offspring diet effect | Offspring diet effect |
|  |  |  |  | OB»C vs C»C | OB»OB vs C»OB | C»OB vs C»C | OB»OB vs OB»C |
| Weight (7wk) | NS | *↑ | * | *↑ | NS | *↑ | *↑ |
| LDC | NS | *↑ | NS | NS | NS | *↑ | *↑ |
| mtDNA copy number | NS | NS | NS | NS | NS | NS | NS |
| MMP | NS | *↑ | NS | $↑ | NS | *↑ | $↑ |
| ROS | NS | *↑ | NS | NS | NS | *↑ | $↑ |
| Distribution | NS | NS | NS | NS | NS | NS | NS |
| Ultrastr. (Total) | *↑ | *↑ | * | $↑ | $↑ | *↑ | NS |
| Dumbbell | NS | *↑ | NS | NS | NS | NS | NS |
| Degenerative | *↑ | *↑ | NS | NS | *↑ | *↑ | NS |
| Elongated | *↑ | NS | NS | NS | NS | NS | NS |
| E^-^dense | *↑ | NS | * | $↑ | NS | $↑ | NS |
| Rose-petal | NS | NS | $ | NS | NS | NS | NS |
| Loose inner membrane | NS | NS | NS | NS | NS | NS | NS |
| ETC CI | ND | ND | ND | ND | ND | ND | ND |
| ETC CII | NS | NS | NS | NS | NS | NS | NS |
| ETC CIII | *↓ | NS | NS | NS | NS | NS | NS |
| ETC CIV | NS | NS | NS | NS | NS | NS | NS |
| ETC CV | *↓ | NS | NS | *↓ | NS | NS | NS |
| Pyruvate | *↓ | *↓ | NS | $↓ | NS | $↓ | *↓ |
| Lactate | $↓ | NS | NS | NS | NS | NS | NS |
|  | | | | | | | |
