# Supplementary material for: The impact of a maternal and offspring obesogenic diet on daughter’s oocyte mitochondrial ultrastructure and bioenergetic responses. Insights from an outbred mouse model
Source: Front Physiol. 2023 Oct 25;14:1288472. doi: 10.3389/fphys.2023.1288472 (PMC10642210; doi:10.3389/fphys.2023.1288472)
Supplement: Supplementary file 2 [file DataSheet1.DOCX]

**Manuscript title: The Impact of a Maternal and Offspring Obesogenic diet on Daughter’s Oocyte Mitochondrial Ultrastructure and Bioenergetic Responses. Insights from an Outbred Mouse Model.**

**Supplementary Information 1: Maternal weight and litter size**

Maternal OB diet significantly increased the weight of the mother at mating (weight at week 7, *P*<0.05). The maternal OB diet significantly increased live body weight from week 3 onwards. Overall body weight trajectory of the mothers (using repeated measures ANOVA) was affected by diet, time and the interaction between both factors (*P*<0.05). The weight of the mother at mating was not correlated with litter size (r = 0.32, *P*>0.05). More detailed information is shown in fig. S1 and table S1. Litter size was not correlated with offspring body weight (r = 0.015, *P*>0.05).


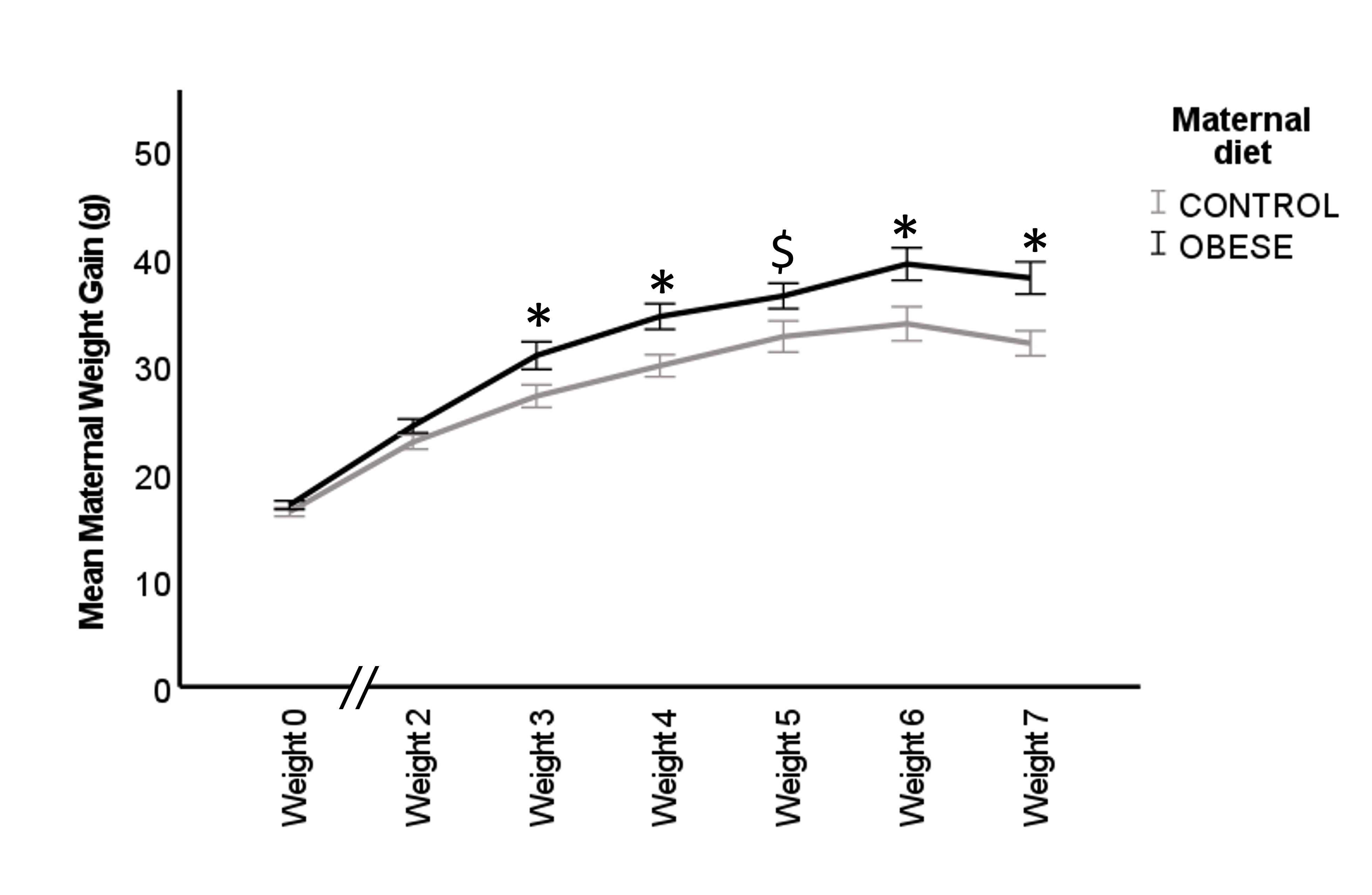


| Fig. S1. Maternal diet effect on maternal weight gain trajectory. The graph shows maternal weight at the start of the trial (Weight 0, weaning of the mothers) until mating (Weight 7) in mothers fed a control (C) or an obesogenic diet (OB). Data are presented as mean±S.E.M. and are derived from 11 C mothers and 15 OB mothers. Significant differences per timepoint (Independent Samples T-test) are indicated with an asterisk (*), tendencies with a dollar sign ($). |
| --- |

| Table S1. Litter characteristics of C-fed or OB-fed mothers and the corresponding correlation with the weight of the mother at the time of mating (7w). Data are represented as mean±S.E.M. and are derived from 11C and 15 OB mothers. |
| --- |
| \|  \| Litter size \| Litter weight \| Pup weight at birth \| \| --- \| --- \| --- \| --- \| \| CONTROL n=11 \| 14.45±0.61 \| 30.26±1.35 \| 2.22±0.20 \| \| OBESE n=15 \| 14.26±0.41 \| 30.15±0.93 \| 2.14±0.07 \| \| r weight F_0_ \| 0.32 \| 0.119 \| -0.059 \| \| *P*-value \| 0.876 \| 0.562 \| 0.775 \| |
|  |
